# Quorum Sensing Coordinates Cooperative Expression of Pyruvate Metabolism Genes To Maintain a Sustainable Environment for Population Stability

**DOI:** 10.1128/mBio.01863-16

**Published:** 2016-12-06

**Authors:** Lisa A. Hawver, Jennifer M. Giulietti, James D. Baleja, Wai-Leung Ng

**Affiliations:** a Department of Molecular Biology and Microbiology, Tufts University School of Medicine, Boston, Massachusetts, USA; b Department of Developmental, Molecular and Chemical Biology, Tufts University School of Medicine, Boston, Massachusetts, USA; c Program in Molecular Microbiology, Sackler School of Graduate Biomedical Sciences, Tufts University, Boston, Massachusetts, USA

## Abstract

Quorum sensing (QS) is a microbial cell-cell communication system that regulates gene expression in response to population density to coordinate collective behaviors. Yet, the role of QS in resolving the stresses caused by the accumulation of toxic metabolic by-products at high cell density is not well defined. In response to cell density, QS could be involved in reprogramming of the metabolic network to maintain population stability. Using unbiased metabolomics, we discovered that *Vibrio cholerae* mutants genetically locked in a low cell density (LCD) QS state are unable to alter the pyruvate flux to convert fermentable carbon sources into neutral acetoin and 2,3-butanediol molecules to offset organic acid production. As a consequence, LCD-locked QS mutants rapidly lose viability when grown with fermentable carbon sources. This key metabolic switch relies on the QS-regulated small RNAs Qrr1-4 but is independent of known QS regulators AphA and HapR. Qrr1-4 dictate pyruvate flux by translational repression of the enzyme AlsS, which carries out the first step in acetoin and 2,3-butanediol biosynthesis. Consistent with the idea that QS facilitates the expression of a common trait in the population, AlsS needs to be expressed cooperatively in a group of cells. Heterogeneous populations with high percentages of cells not expressing AlsS are unstable. All of the cells, regardless of their respective QS states, succumb to stresses caused by toxic by-product accumulation. Our results indicate that the ability of the bacteria to cooperatively control metabolic flux through QS is critical in maintaining a sustainable environment and overall population stability.

## IMPORTANCE

Our work reveals a novel role for *Vibrio cholerae* quorum sensing (QS) in relieving the stresses caused by toxic metabolite accumulation when the population becomes crowded through metabolic reprogramming. QS enables *V. cholerae* switching from a low cell density energy-generating metabolism that is beneficial to individuals at the expense of the environment to a high cell density mode that preserves environmental habitability by sacrificing individual fitness. This cooperative switch provides a stable environment as the common good in maintaining the stability of the community. However, the common good can be exploited by uncooperative mutants that pollute the environment, causing population collapse. Our findings provide insights into the metabolic stress response of a major human pathogen, with implications for our understanding of microbial social biology and cooperation from an ecological and evolutionary perspective.

## INTRODUCTION

Many microbial collective processes are ineffective if performed by a single bacterial cell acting alone. To successfully execute these tasks, bacteria employ a cell-to-cell communication process called quorum sensing (QS), in which they produce and detect extracellular signaling molecules called autoinducers to monitor cell population density, to coordinate gene expression within a group, and to act in unison (1–4). Moreover, increasing evidence indicates that QS is linked to regulation of metabolic homeostasis and readjustment (reviewed in reference 5). Since QS is employed to monitor cell density, it is possible that QS-dependent metabolic reprogramming is used to cope with the stress caused by toxic by-product accumulation due to overpopulation. Yet, the role of metabolic reprogramming in the stability of a QS bacterial population and the link between metabolism and the social behavior of a microbial community are not clear.

**FIG 1:**
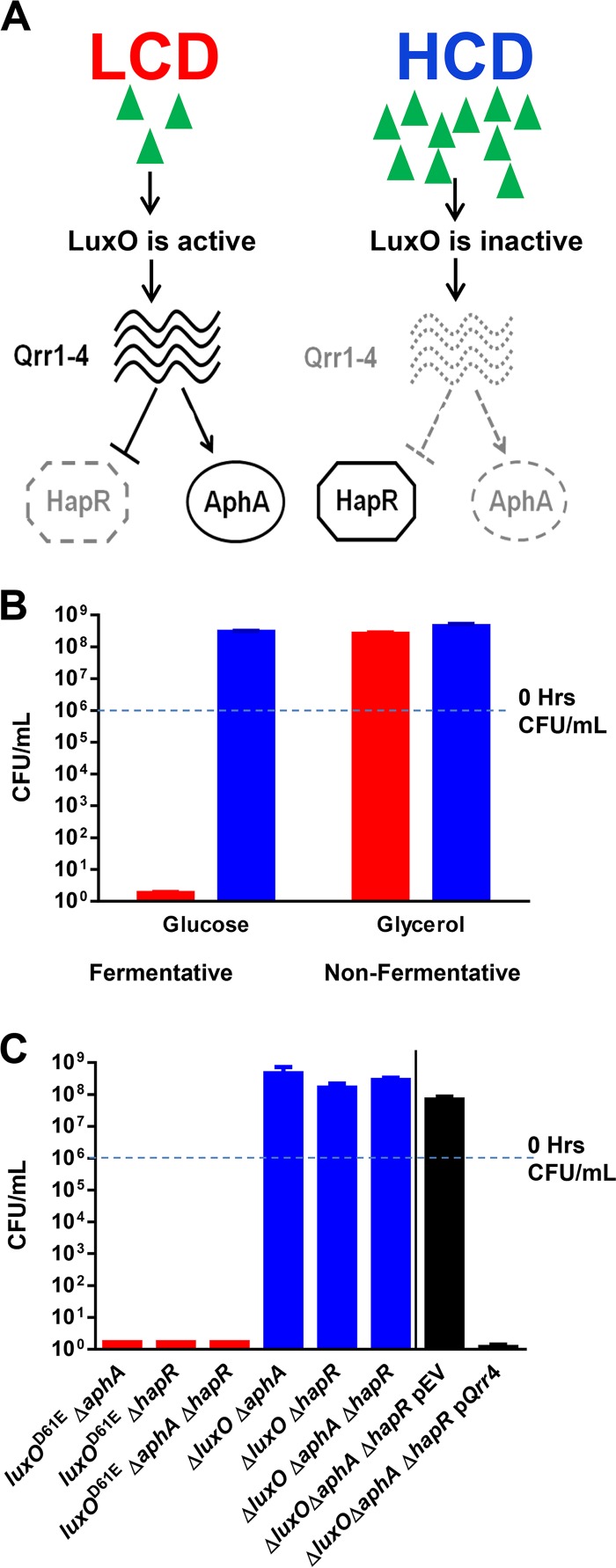
The roles of QS in resolving fermentative stress in *V. cholerae*. (A) Simplified view of the *V. cholerae* QS circuit. (B) Viability of *luxO*^D61E^ Δ*vpsL* (red bars) and Δ*luxO* Δ*vpsL* (blue bars) mutant cells at 72 h of growth under fermentative and nonfermentative conditions. (C) Viability of *luxO*^D61E^ (red bars) and Δ*luxO* (blue bars) mutants with additional QS mutations and the Δ*luxO* Δ*aphA* Δ*hapR* mutant strain carrying an empty plasmid or a plasmid overexpressing Qrr4 (black bar) at 72 h of growth under fermentative conditions. All strains are Δ*vpsL* mutants. The limit of detection at 72 h is 2 cells/ml of culture. The values shown are averages of at least three replicates. Error bars denote the SEM.

*Vibrio cholerae*, the causative agent of the disease cholera, uses QS to control a variety of group behaviors, including virulence factor production, biofilm formation, type VI secretion, and natural competence development (6–15). The QS response of *V. cholerae* is controlled by a small-RNA (sRNA)-based regulatory system (8, 16–19). At low cell density (LCD), when extracellular autoinducer levels are low, the key QS regulator LuxO is activated by phosphorylation through the action of four parallel signaling pathways (11, 15, 20). Consequently, phosphorylated LuxO (LuxO~P) activates the transcription of the genes encoding four regulatory sRNAs called Qrr1-4 (18). Qrr1-4 function redundantly to activate the translation of the AphA regulator and inhibit the translation of the HapR regulator (18–22) (Fig. 1A). At high cell density (HCD), when autoinducer levels are high, LuxO is dephosphorylated and becomes inactive. Thus, transcription of the genes for Qrr1-4 terminates and, hence, HapR is produced but AphA is not (Fig. 1A). Reciprocal production of these two regulators is believed to be crucial for the global control of QS behaviors in *V. cholerae* (21). Furthermore, Qrr sRNAs also directly control the expression of a number of targets, including feedback regulation of LuxO, a diguanylate cyclase (VCA0939), and components of a type VI secretion apparatus (17, 23–25). Yet, our understanding of the role of QS in *V. cholerae* biology is incomplete. The *V. cholerae* QS regulon is estimated to include over 100 genes (11, 21, 26, 27), significantly exceeding the number of genes related to the aforementioned processes. Thus, the QS regulon could have a broader impact on *V. cholerae* survival and fitness inside and/or outside the host.

Here we report that conversion of the central carbon metabolite pyruvate into different fermentative end products (organic acid, acetoin/2,3-butanediol [2,3-BD]) is dictated by the Qrr1-4 sRNAs in *V. cholerae*. Moreover, we provide evidence that organic acid production and acetoin/2,3-BD production are two contrasting social behaviors. The former pathway increases individual fitness by exploiting a common inhabitable environment, while the latter pathway benefits the group by sacrificing individual fitness. Massive invasion of an otherwise cooperative population by uncooperative cells that make organic acids constitutively leads to pollution of the otherwise habitable environment and kills the entire population. Our results indicate that the ability of bacteria to cooperatively control metabolic flux through QS is critical in maintaining a sustainable environment and overall population stability.

## RESULTS

### LCD QS mutants are sensitive to sugar fermentative stress.

To begin to learn more about the physiological roles of *V. cholerae* QS, we tested if growth is adversely affected in mutants genetically locked in the LCD or HCD QS state. Here we define the *luxO*^D61E^ mutant as the LCD-locked strain since this *luxO* allele renders LuxO constitutively active by mimicking the active form of the regulator, resulting in constant production of Qrr1-4 sRNAs (Fig. 1A) (15, 17, 18). In contrast, we used the Δ*luxO* mutant as the HCD-locked strain since no Qrr1-4 sRNA is produced in the absence of LuxO (Fig. 1A) (15, 17, 18). Since carbon utilization influences many aspects of the *V. cholerae* life cycle (28–38), we first monitored the growth of these two QS mutants in M9 medium supplemented with amino acids and different carbon sources and monitored viable cell counts for at least 72 h. When no fermentable carbon source was present (e.g., medium with glycerol), the viable cell counts of both QS-locked mutants increased comparably and they maintained viability (Fig. 1B; see Fig. S1A in the supplemental material). However, when a fermentable sugar (e.g., glucose or *N*-acetylglucosamine [GlcNAc]) was present, the viable cell counts of both mutants increased during the first 24 h and the HCD-locked mutant maintained viability thereafter but the LCD-locked mutant lost viability rapidly over the next 48 h (Fig. 1B; see Fig. S1B and C). We introduced a Δ*vpsL* mutation (39–41) into our strains to rule out the potential contribution of biofilm formation. There was no difference between the observed growth phenotypes of these *vpsL* mutant strains and their *vpsL*^+^ counterparts (see Fig. S1). *V. cholerae* strains carrying the wild-type (WT) *luxO* allele, similar to the HCD-locked mutant, were able to grow and maintain viability under these conditions (see Fig. S2 in the supplemental material). The difference in growth between these two QS mutants under this specific condition suggests that one or more metabolic pathways involved in carbon catabolism are controlled by QS and misregulated in the LCD-locked QS mutant.

### Sugar fermentative stress is mediated by Qrr sRNAs independently of other QS regulators.

The LCD QS regulator AphA has been shown to repress the transcription of the *alsDSO* operon involved in acetoin biosynthesis (42). Mutants unable to synthesize acetoin are sensitive to glucose fermentation (42–44). Thus, the loss of viability could be attributed to constitutive production of AphA by the LCD-locked mutants, resulting in constant repression of the *alsDSO* operon. When we deleted the *aphA* gene from the LCD-locked mutant, however, the sensitivity to fermentative stress was maintained (Fig. 1C; see Fig. S3 in the supplemental material). Since HapR represses AphA expression, we predicted that a *hapR* deletion might diminish the resistance of the HCD-locked mutant to fermentative stress. However, an HCD-locked mutant lacking HapR remained resistant to fermentative stress. Similarly, LCD-locked mutants missing both AphA and HapR were still sensitive, while HCD-locked mutants missing both AphA and HapR remained resistant to fermentative stress (Fig. 1C; see Fig. S3). When Qrr4 was overexpressed ectopically in a Δ*luxO* Δ*aphA* Δ*hapR* triple mutant, these cells became sensitive to fermentative stress (Fig. 1C). These results indicate that the sensitivity to fermentative stress observed in the LCD-locked QS mutants is Qrr1-4 mediated and independent of the two QS regulators AphA and HapR. None of the *V. cholerae* Qrr1-4-regulated genes is known to be involved in resolving fermentative stress (24, 25). Our results suggest that a new physiological role for Qrr1-4 exists outside their known roles in QS gene regulation.

### Cell death in LCD-locked mutants is caused by unregulated excretion of organic acids.

**FIG 2:**
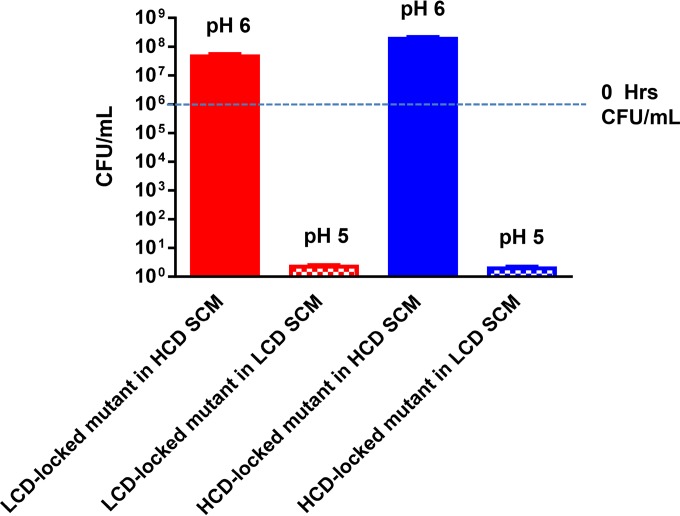
The LCD supernatant is toxic. The LCD-locked mutant (*luxO*^D61E^ Δ*aphA* Δ*hapR* Δ*vpsL*) (red) and the HCD-locked mutant (Δ*luxO* Δ*aphA* Δ*hapR* Δ*vpsL*) (blue) were measured at 72 h of growth in HCD SCM (solid bars) or LCD SCM (checkered bars). The blue dotted line indicates the initial cell viability of each strain at 0 h. The limit of detection at 72 h is 2 cells/ml of culture. The values shown are averages of at least three replicates. Error bars denote the SEM.

**FIG 3:**
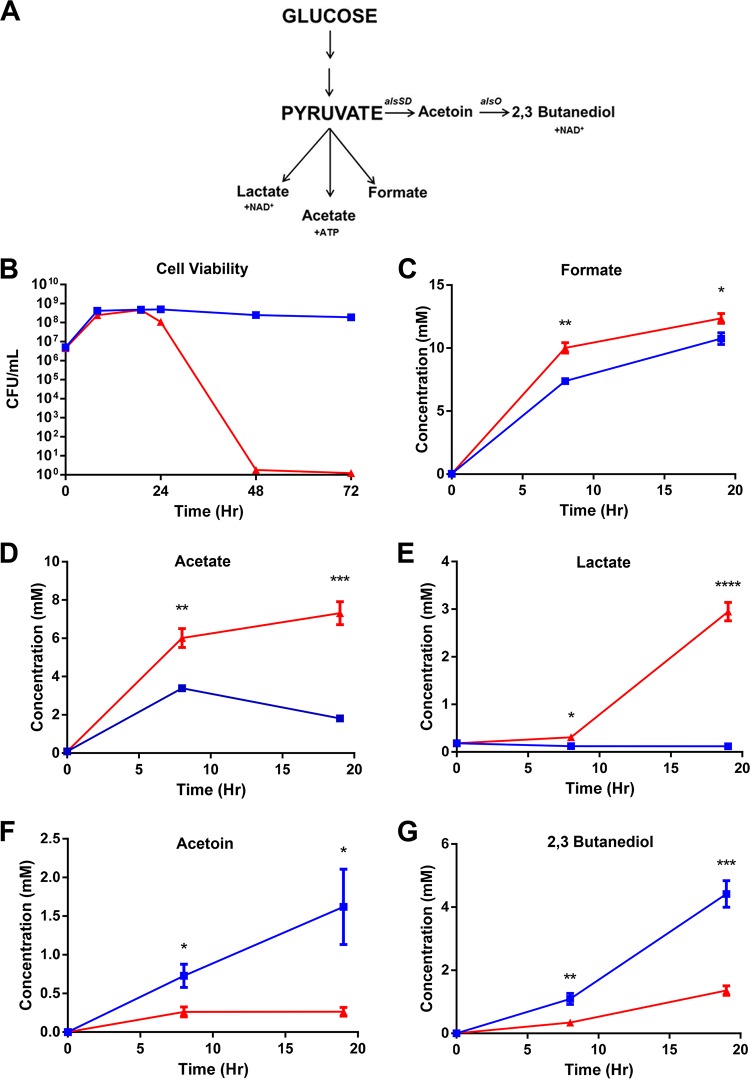
Extracellular metabolite profiles of QS mutants grown under fermentative conditions. (A) Simplified view of glycolysis and pyruvate metabolism in *V. cholerae*. (B) Viability of LCD-locked (*luxO*^D61E^ Δ*aphA* Δ*hapR* Δ*vpsL*; red solid triangles) and HCD-locked (Δ*luxO* Δ*aphA* Δ*hapR* Δ*vpsL*; blue solid squares) cells. Concentrations (millimolar) of formate (C), acetate, (D), lactate (E), acetoin (F), and 2,3-BD (G) in LCD-locked (red solid triangles) and HCD-locked (blue solid squares) mutants. *P* values (Student’s *t* test): *, *P* < 0.05; **, *P* < 0.01; ***, *P* < 0.001; ****, *P* < 0.0001. The values shown are averages of at least three replicates. Error bars denote the SEM.

To test if the LCD-locked mutants accumulate toxic intermediates intracellularly during glucose fermentation or if the toxicity is due to changes in the external milieu, we harvested spent culture medium (SCM) from LCD-locked mutants grown in the presence of glucose and tested if LCD SCM is toxic to the two QS-locked mutants. We found that both strains failed to maintain viability in LCD SCM (Fig. 2, checkered bars; see Fig. S4 in the supplemental material), suggesting that the SCM contains one or more toxic substances excreted by the LCD-locked mutant. It also indicates that the HCD-locked mutant does not have a mechanism to detoxify these substances. In contrast, both strains grew and maintained viability over time in SCM prepared from the HCD-locked mutant (Fig. 2, solid bars; see Fig. S4), suggesting that HCD-locked mutants produce none or low levels of these toxic by-products. After 72 h of growth, the pH of LCD SCM was ~5, whereas that of HCD SCM was ~6 (Fig. 2), consistent with the hypothesis that the toxicity is due to acidification of the external milieu during sugar fermentation by the LCD-locked mutants. In *V. cholerae*, glucose fermentation leads to several end products, some of which are acidic (Fig. 3A). Glucose is catabolized to pyruvate through glycolysis. During fermentation, pyruvate is in flux among several pathways. First, pyruvate can be fed to three different organic acid production pathways, leading to excretion of lactate, acetate, and formate (Fig. 3A). The lactate pathway provides a mechanism of NAD^+^ regeneration, while ATP is generated through the acetate pathway. Pyruvate can also be converted to the neutral molecules acetoin and 2,3-BD with regeneration of NAD^+^ and liberation of CO_2_ (Fig. 3A). Although this pathway does not neutralize organic acids, it directs the flux of pyruvate away from organic acid production while still allowing NAD^+^ regeneration, albeit without ATP production. Since the pH of LCD-locked SCM is acidic, we reasoned that the LCD-locked mutant is producing more extracellular organic acids than the HCD-locked mutant. First, we focused on SCM from two QS-locked mutants (without AphA and HapR) grown with glucose as the sole fermentable sugar. We analyzed the metabolite profiles of the two QS-locked mutants up to 19 h after inoculation to avoid measurement of metabolites that might leak from dead cells (Fig. 3B). By using an unbiased nuclear magnetic resonance (NMR) metabolomic approach (45), more than 20 extracellular metabolites could be detected and quantified (see Table S2 in the supplemental material). No significant difference between the two QS-locked mutants was detected in the concentrations of the majority of these metabolites at multiple time points (see Table S2). However, the concentrations of organic acids derived from pyruvate (i.e., formate, acetate, and lactate) were significantly higher in LCD SCM than in HCD SCM (Fig. 3C to E; see Table S2). Moreover, extracellular levels of the neutral molecules acetoin and 2,3-BD in HCD SCM were significantly higher than those in LCD SCM (Fig. 3F and G; see Table S2). The extracellular metabolome profiles of the WT and a *luxO*^+^ Δ*aphA* Δ*hapR* mutant were similar to the profiles obtained from the respective isogenic HCD-locked mutants (see Table S2 and Fig. S5 in the supplemental material). Together, these results suggest that *V. cholerae* uses QS, in particular Qrr1-4 sRNAs, to control central carbon metabolism by altering pyruvate flux in favor of organic acid production at LCD and then switching to neutral molecule production at HCD, presumably to regulate the potential overproduction of toxic metabolic by-products. Consistent with our data, the addition of amounts of formic acid, acetic acid, and lactic acid equivalent to those detected in LCD SCM to a culture of the HCD-locked QS mutant was sufficient to kill all of the cells in the population (see Fig. S6 in the supplemental material). In contrast, LCD-locked mutant viability was maintained by buffering the culture medium to a neutral pH (see Fig. S6).

### Uncoupling of Qrr1-4 regulation of *alsS* suppresses glucose sensitivity in LCD-locked mutants.

**FIG 4:**
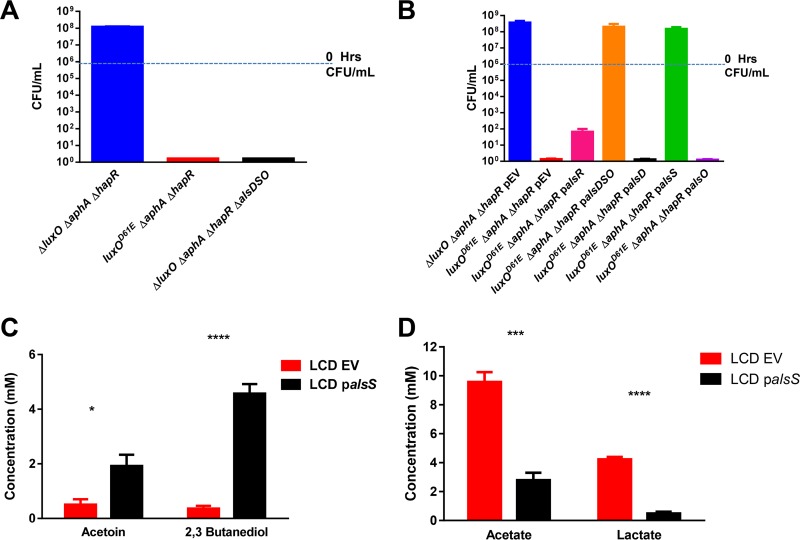
AlsS misregulation causes fermentation stress in QS mutants. (A) Cell viability of the HCD-locked strain (Δ*luxO* Δ*aphA* Δ*hapR* Δ*vpsL*; blue bar), the LCD-locked strain (*luxO*^D61E^ Δ*aphA* Δ*hapR* Δ*vpsL*; red bar), and the HCD-locked strain carrying the Δ*alsDSO* deletion (black bar) at 72 h of growth under fermentative conditions. (B) Cell viability at 72 h of the HCD-locked strain (blue bar), the LCD-locked strain (red bar), and the LCD-locked strain harboring the *als* overexpression plasmids p*alsR* (pink bar), p*alsDSO* (orange bar), p*alsD* (black bar), p*alsS* (green bar), and p*alsO* (purple bar). (C and D) Extracellular concentrations of selected metabolites of the LCD-locked strain (red bars) and the LCD-locked strain with p*alsS* (black bars) grown under fermentative conditions. Millimolar concentrations are shown, and samples were taken at 19 h of growth. The values shown are averages of at least three replicates. Error bars denote the SEM. *P* values (Student’s *t* test): *, *P* < 0.05; ***, *P* < 0.001; ****, *P* < 0.0001. EV, empty vector.

We then tested if deletion of the acetoin/2,3-BD biosynthetic operon *alsDSO* (42) from the HCD-locked QS mutant (lacking AphA and HapR) would cause the strain to become sensitive to growth in glucose. Indeed, the *alsDSO* deletion caused the strain to become sensitive to fermentative stress, similar to the LCD-locked mutant (Fig. 4A). In contrast, overexpression of the *alsDSO* operon under the control of a heterologous promoter and ribosome binding site (RBS) in the LCD-locked mutant (lacking AphA and HapR) relieved glucose sensitivity (Fig. 4B). Strikingly, overexpression of *alsS* alone, but not overexpression of *alsD, alsO*, or* alsR*, encoding the positive transcriptional regulator of the *alsDSO* operon (42), also suppressed the glucose sensitivity of the LCD-locked mutant (Fig. 4B). Overproduction of AlsS alone also caused the extracellular concentrations of acetoin and 2,3-BD to increase in an LCD-locked strain, while acetate and lactate concentrations decreased significantly (Fig. 4C and D). These results are consistent with a model in which production of Qrr1-4 in LCD-locked QS mutants reduces *alsS* expression, leading to sensitivity to fermentative stress.

### Qrr sRNAs regulate *alsS* expression post-transcriptionally.

**FIG 5:**
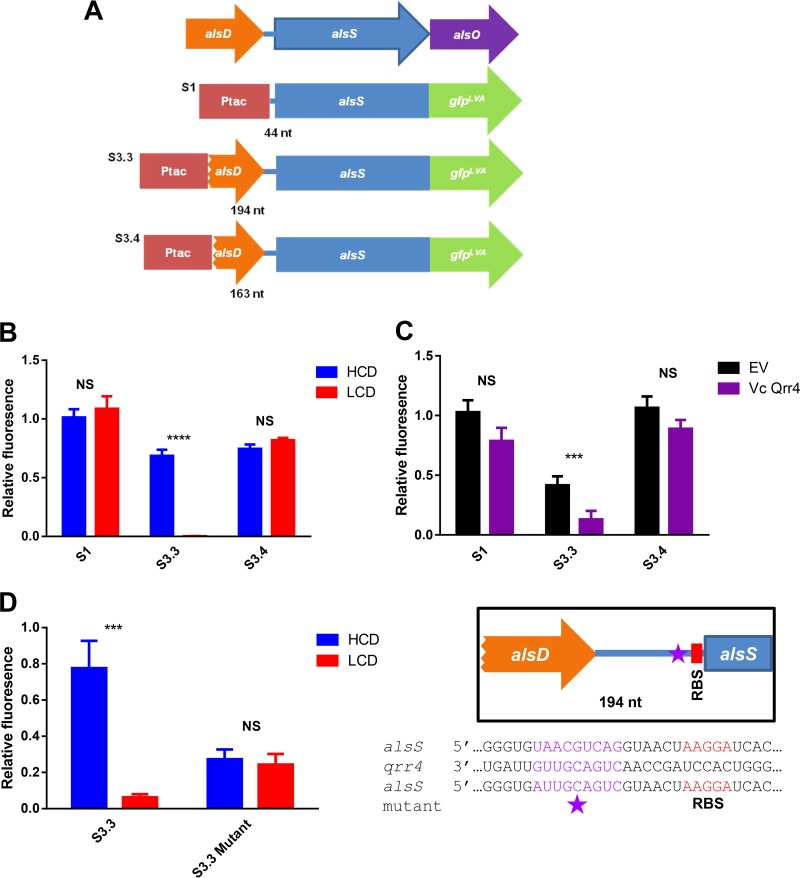
Qrr1-4 regulate *alsS* translation. (A) Depiction of the *alsDSO* operon and the different GFP translational fusions used in this study. nt, nucleotides. (B) Relative GFP fluorescence of different GFP translational fusions in *V. cholerae* HCD-locked (Δ*luxO* Δ*aphA* Δ*hapR* Δ*vpsL*; blue bars) and LCD-locked (*luxO*^D61E^ Δ*aphA* Δ*hapR* Δ*vpsL*; red bars) strains. Each bar is normalized to the median FLU value of the HCD strain harboring the S1 plasmid. (C) Relative GFP fluorescence of different GFP translational fusions in *E. coli* strains either carrying an empty vector (EV) plasmid (black bars) or a plasmid containing rhamnose-induced *V. cholerae qrr4* (purple bars). Each bar is normalized to the median FLU value of the *E. coli* strain carrying the S1 and EV plasmids. (D) Relative GFP fluorescence of GFP translational fusion S3.3 and S3.3 harboring nine consecutive mutations in a predicted Qrr binding site in HCD-locked (blue bars) and LCD-locked (red bars) *V. cholerae* strains. Each bar is normalized to the median FLU value of the HCD strain harboring the S1 plasmid. Highlighted are the location of a hypothetical Qrr binding site in *alsS* (purple star), the sequence of the mutant construct, and the predicted RBS. The values shown are averages of at least three replicates. Error bars denote the SEM. *P* values (Student’s *t* test): ***, *P* < 0.001; ****, *P* < 0.0001. NS, no statistical significance.

To determine the mechanism by which Qrr1-4 reduces *alsS* expression, we constructed AlsS-GFP translational reporters by fusing an isopropyl-β-d-thiogalactopyranoside (IPTG)-inducible P_*tac*_ promoter to various lengths of the *alsS* 5′ upstream region plus the *alsS* coding region, followed by the gene encoding an unstable version of green fluorescent protein (GFP) (Fig. 5A). We introduced these constructs into both LCD-locked and HCD-locked QS strains lacking HapR and AphA and measured the GFP fluorescence generated from these reporters per cell. The first construct, S1, which contains 44 bp upstream of the start codon of *alsS* with the predicted RBS (Fig. 5A), produced similar amounts of fluorescence in both QS-locked mutants (Fig. 5B). In contrast, LCD-locked mutants harboring the construct S3.3, which contains an additional 150-bp region that extends into the *alsD* gene (Fig. 5A), produced significantly less fluorescence than the HCD-locked mutants carrying the same constructs (Fig. 5B). Isogenic strains carrying a WT *luxO* allele harboring these constructs produced levels of fluorescence similar to those of the HCD-locked mutants (see Fig. S7 in the supplemental material).

To determine if Qrr1-4 directly interacts with the mRNA of *alsS*, we introduced the S1 and S3.3 reporters into a heterologous *Escherichia coli* host (21) that does not normally have the *V. cholerae* QS circuit or the *alsS* gene. Instead, *V. cholerae qrr4* was produced ectopically from a plasmid by using a rhamnose-inducible promoter. Similar GFP levels were detected in strains carrying the S1 construct, whether Qrr4 was overexpressed or not (Fig. 5C). In contrast, GFP expression in the cultures carrying the S3.3 reporter was significantly reduced when Qrr4 was overproduced (Fig. 5C). Although we could not rule out the possibility that Qrr4 has an impact on an unknown *E. coli* factor that could regulate *alsS* expression, our results suggest that Qrr sRNAs likely repress *alsS* expression through a direct mechanism. We also performed quantitative reverse transcription-PCR to determine the *alsS* and *gfp* transcript levels in different *E. coli* reporter cultures with and without Qrr4 overexpression. The *alsS* and *gfp* transcript levels were comparable in all cultures (data not shown), arguing against a role for Qrr sRNAs in transcriptional termination.

We then made a 5′ truncation of the S3.3 construct (Fig. 5A). The resulting construct, S3.4, is 31 bp shorter than the S3.3 construct. We found that repression of GFP production by Qrr sRNAs was abolished in the S3.4 construct in the LCD-locked *V. cholerae* strain (Fig. 5B; see Fig. S7). This region contains the signature ARN/AAN Hfq binding motif (46), suggesting that Qrr sRNAs require Hfq to repress *alsS* expression. Indeed, an LCD-locked mutant with a Δ*hfq* mutation is not sensitive to fermentative stress. In addition, we used an sRNA binding site prediction program, IntaRNA (47, 48), to locate a potential Qrr binding site within the *alsS* 5′ untranslated region (UTR). The program predicted a consecutive nine-nucleotide putative Qrr binding site just six nucleotides 5′ to the predicted RBS of *alsS* (Fig. 5D). Mutation of this site in the S3.3 construct lowered the overall GFP production level but abolished the repression by Qrr sRNAs in the *V. cholerae* LCD-locked strain (Fig. 5D), suggesting that Qrr sRNAs could potentially bind to this site and occlude the ribosome from translating the *alsS* transcript. However, when we introduced this mutated construct into the heterologous *E. coli* host, we could not detect any GFP production, regardless of whether Qrr4 was expressed or not. Similarly, mutation of this site in the S3.4 construct abolished GFP production. The lack of GFP expression in these mutated constructs in *E. coli* could be due to poor recognition of these sequences by the *E. coli* ribosome. Together, our results strongly suggest that Qrr sRNAs repress *alsS* expression through a posttranscriptional mechanism.

### Community stability requires coordinated control of pyruvate metabolism.

**FIG 6:**
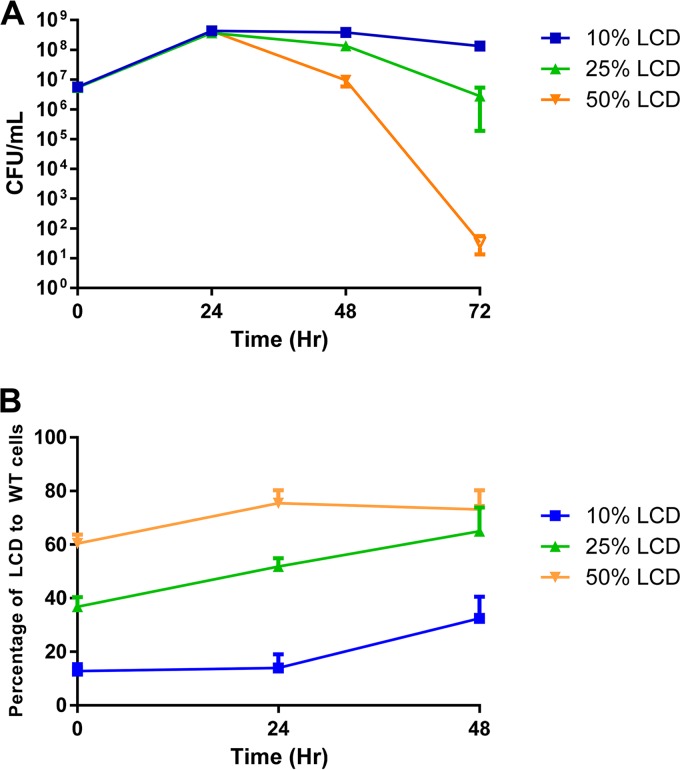
Population stability requires cooperative expression of carbon metabolic genes. (A) Cell viability under fermentative conditions. (B) Percentage of LCD-locked cells present in the overall culture over time. The coculture experiments commenced with different ratios of LCD-locked (*luxO*^D61E^ Δ*vpsL*) QS mutant to WT (Δ*vpsL*) bacteria as shown. The limit of detection at 72 h is 1 cell/ml of culture (open symbol). The values shown are averages of at least three replicates. Error bars denote the SEM.

QS is generally assumed to coordinate cooperative behaviors. Why, then, is it used to regulate pyruvate flux and determine the level of expression of acetoin/2,3-BD production? Organic acid production pathways can be viewed as beneficial to individual bacteria because they generate one ATP per pyruvate consumed; however, this process also exploits the common environment that the group inhabits, resulting in acidification of the extracellular milieu. In contrast, the acetoin/2,3-BD production pathway regenerates NAD^+^ but does not lower the environmental pH, making it more appropriate for a relatively dense population, and can be considered a “cooperative” behavior in which each individual sacrifices its own fitness to maintain a habitable environment. On the basis of these assumptions, we asked if a cooperative population would form a stable community and if “selfish” individuals have better fitness when present in small numbers but would cause a tragedy of the commons when they become predominant in the population (49–52). We mixed different percentages of LCD-locked mutant (*luxO*^D61E^) and WT *V. cholerae* cells together and cocultured the two strains in the presence of glucose (Fig. 6A). When the initial percentage of the LCD-locked mutant in the population was ~10%, the entire group remained stable and the total viable cell count after 72 h of growth was similar to that of a population composed entirely of 100% WT cells (Fig. 6A; see Fig. S8A and B in the supplemental material). The percentage of the LCD-locked mutant in this group was maintained at approximately 10% for the first 24 h and increased to ~30% at 48 h, but it did not take over the population (Fig. 6B). The increase in the abundance of LCD-locked mutants in this group suggests that the strain has higher individual fitness than the WT, likely because of an increased ATP yield generated from pyruvate conversion to acetate. In a mixed population containing ~25% LCD-locked mutants and 75% WT bacteria, a slight decrease in the overall population viability was detected at both 48 and 72 h, concurrent with an ~2-fold increase in the percentage of LCD-locked mutants in the population. When 50% of the LCD-locked mutant and 50% of the WT bacteria were cocultured at the outset of the experiment, massive cell death resulting in total population collapse was observed (Fig. 6A). The percentage of LCD-locked mutants in this group increased only slightly (<2-fold), but they represented more than half of the population (Fig. 6B). Rapid killing of the entire population was observed when LCD-locked QS mutants were present at even higher percentages (see Fig. S8A and B). Similar results were obtained when *luxO*^D61E^ mutants were cocultured with Δ*luxO* mutants (see Fig. S8C and D). Together, these results suggest that the choice between organic acid production and acetoin/2,3-BD production has to be coordinated and cooperative in order to maintain long-term population stability, and QS has evolved to minimize the danger to the population by preventing uncontrolled acidification of the shared environment.

## DISCUSSION

Here we show that Qrr sRNAs play a unique role in maintaining population stability by coordinating pyruvate metabolism. While QS has been shown to affect carbon metabolism (acetate switch) in other *Vibrio* species (53), we illustrate here a new role for Qrr1-4 in metabolic regulation through coordinating the flux of the key carbon metabolite pyruvate at different cell densities. Our new data suggest that, in addition to AphA, a QS regulator that has been previously shown to repress the transcription of the *alsDSO* operon (42), Qrr1-4 sRNAs ensure that acetoin and 2,3-BD are not produced by blocking *alsS* translation. Furthermore, (p)ppGpp (a product of the stringent response) positively regulates the transcription of the *alsDSO* operon in *V. cholerae* and (p)ppGpp-null mutants lose viability because of fermentative stress (43). Our results and those of others, taken together, suggest that multiple layers of regulation are employed to control acetoin/2,3-BD production in *V. cholerae*. Our new results also expand the repertoire of sRNAs that regulate carbon metabolism in bacteria (54–58).

Acetoin and 2,3-BD production has been shown to be activated by QS in other species besides *V. cholerae* (59, 60), but it is unclear what the evolutionary advantage of employing QS for such regulation is. Although it is generally believed that QS is used to coordinate and modulate cooperative behaviors at the population level, metabolic flux control is not usually perceived as a social behavior. We propose here, however, that organic acid production and neutral metabolite production can be considered two contrasting social traits. Our data suggest that QS allows cells to maximize individual fitness when the cell number in the population is low, as reflected by the increase in the proportion of LCD-locked strains in mixed cultures with WT or HCD-locked strains. At LCD, there is little incentive for cells to make acetoin and 2,3-BD, as ATP is not produced through this pathway. However, continuous production of organic acids could lead to acidification of the extracellular milieu, and *V. cholerae* is known to have low tolerance for a low pH (61). Thus, *V. cholerae* has evolved to sacrifice the ability to produce ATP via the acetate synthesis pathway and switch pyruvate flux to the production of the neutral molecules acetoin and 2,3-BD to maintain a sustainable and well-preserved environment to prevent population collapse at HCD. Interestingly, although many *V. cholerae* strains carry the *alsDSO* operon, production of acetoin and 2,3-BD is observed mainly in the *V. cholerae* O1 El Tor serotype, the current pandemic strain (44). Metabolic slowing due to QS-dependent nutrient uptake restriction has also been observed in *Burkholderia glumae* (62).

A similar scenario was observed in some *Burkholderia* species in which QS-positive bacteria produce oxalate, an extracellular metabolite that neutralizes the ammonia released during amino acid catabolism (63). Thus, QS in *Burkholderia* is used to anticipate overcrowding and promote bacterial survival, and QS mutants that fail to produce oxalate encounter massive and rapid population crashes (63). However, unlike the study here, oxalate production and amino acid catabolism in *Burkholderia* do not involve common precursor metabolites. Thus, these two pathways likely are not mutually exclusive and it is unclear what the fitness cost is for cooperative QS-positive cells to produce oxalate as a benefit to the group (63).

While QS seems to be an effective mechanism to enable cooperation in a population to cope with metabolic stresses due to increasing cell density, this collective behavior can be exploited by social cheaters, which are noncooperative individuals that take advantage of common goods. In many cases, the common goods are extracellular enzymes (49, 64, 65) or shared chemical molecules (e.g., rhamnolipid surfactant) (66, 67) to which cheaters can gain access without their own contribution. In our case, we consider that the “common goods” are not the extracellular metabolites acetoin and 2,3-BD, since these compounds do not neutralize the organic acids that accumulate during growth. Instead, the shared resource is the extracellular environment that both cheaters and cooperators inhabit. We show that the introduction of large quantities of LCD-locked QS mutants (cheaters) leads to a tragedy of the commons (i.e., rapid deterioration of the shared environment) and causes massive population killing.

While LCD-locked QS mutants have been found in natural settings (12, 68, 69), their occurrence is restricted and could be due to selection during growth in laboratories. Indeed, most of the *V. cholerae* strains recently isolated from patient stool samples appear to be QS positive (70), as determined by their natural transformability, a trait that is strictly dependent on a functional QS system. It is possible that rapid killing by fermentative stress is sufficient to eliminate uncooperative subpopulations (i.e., QS-negative LCD-locked mutants), especially in a structurally complex environment where diffusion is limited (71, 72). Alternatively, bacteria have evolved several mechanisms to curb social cheating, including spatial structuring, policing, metabolic constraint, and metabolic prudence (49, 64–66, 71, 73). *V. cholerae* may have evolved to use one or more of these previously described mechanisms to enforce cooperation within the population, and these sustainable bacteria eventually outcompete those that kill themselves with toxic waste.

## MATERIALS AND METHODS

### Strains, plasmids, and culture conditions.

All of the *V. cholerae* strains used in this study were derived from C6706str2, a streptomycin-resistant isolate of C6706 (O1 El Tor) (74). *E. coli* S17-1 λpir was used as a host for plasmids for mating into *V. cholerae* and for *E. coli* GFP assays. All of the strains used in this study are described in Table S1 in the supplemental material. *V. cholerae* and *E. coli* cultures were grown overnight with aeration in Luria-Bertani (LB) broth at 30°C. Cell viability assays were carried out in static culture at 30°C in 1× M9 medium (25.2 mM Na_2_HPO_4_, 22 mM KH_2_PO_4_, 8.56 mM NaCl, 2 mM MgSO_4_, 0.1 mM CaCl_2_) with 0.2% Casamino acids. As a carbon source, 0.5% glucose, 0.5% GlcNAc, or 0.5% glycerol was added. When appropriate, the medium was supplemented with streptomycin (100 μg/ml), ampicillin (100 μg/ml), kanamycin (100 μg/ml), and polymyxin B (50 U/ml).

### DNA manipulations and mutant construction.

All DNA manipulations were performed by using standard procedures as previously described (15). The oligonucleotide sequences used for PCR and sequencing reactions will be provided upon request. Deletions were introduced into the *V. cholerae* genome by allelic exchange by using the suicide vector pKAS32 as previously described (75). Point mutations were introduced by using the QuikChange site-directed mutagenesis kit (Agilent). Open reading frames of genes of interest were PCR amplified with a heterologous RBS and cloned into vector pEVS143 (76) digested with AvrII and BamHI for overexpression. For unstable GFP translational reporters, we first replaced the gene encoding GFP in pEVS143 (76) with an unstable version obtained from pSLS3 (77). Different *alsS* fragments (5′ UTR with the whole *alsS* reading frame without the stop codon) were cloned immediately upstream of the gene encoding unstable GFP without the start codon. Overexpression of *V. cholerae* Qrr4 was achieved by inserting the *qrr4* gene by using Gibson Assembly (NEB) into pRHA109 that contains a rhamnose-inducible P_*rhaB*_ promoter (78). All mutant strains were confirmed by sequencing.

### Cell viability assays.

Overnight cultures were diluted 1,000× and inoculated into M9 minimal medium with 0.2% Casamino acids plus the specified carbon source. Assays were carried out statically at 30°C for at least 72 h. Every 24 h, serial dilutions of each sample were plated to determine viability. Limits of detection are indicated in the figure legends.

### SCM toxicity.

LCD-locked (*luxO*^D61E^ Δ*aphA* Δ*hapR* Δ*vpsL*) and HCD-locked (Δ*luxO* Δ*aphA* Δ*hapR* Δ*vpsL*) QS mutants were grown under fermentative conditions as described above for 72 h. At 72 h, the cells were centrifuged at 3,000 rpm for 15 min. The supernatant was filtered and inoculated with fresh overnight cultures of LCD- and HCD-locked QS mutants diluted 1,000×. Cells were allowed to grow statically at 30°C for 72 h, with an aliquot plated every 24 h to measure cell viability. A universal indicator was used to record the pH at each time point (Fluka Analytical). For assays in which organic acids were added back to determine cell viability, we determined the concentrations of organic acids at 8, 19, 24, 48, and 72 h by NMR metabolomics and added back the total amounts of lactate, acetate, and formate found in LCD SCM at these time points to the HCD culture. For the LCD neutralization experiment, Tris base was added to the SCM until the pH reached 7.2, and then the neutralized SCM was inoculated with fresh LCD cells and cells were plated to determine overall viability at 0, 24, 48, and 72 h.

### NMR metabolomics.

Extracellular metabolite analysis was done with supernatants taken from the growth of LCD (*luxO*^D61E^ Δ*aphA* Δ*hapR* Δ*vpsL*) and HCD (Δ*luxO* Δ*aphA* Δ*hapR* Δ*vpsL*) QS mutants and an isogenic Δ*aphA* Δ*hapR* Δ*vpsL* mutant strain grown under fermentative conditions as described above. Cells were centrifuged at 3,000 rpm for 15 min. Supernatants were removed from the pellets, sterile filtered through a syringe filter (0.2 µM PES), and stored at −20°C until NMR analysis. Five hundred microliters of the supernatants was transferred to 7-in., 600-MHz NMR tubes (Wilmad). A final concentration of 0.180 mM 4,4-dimethyl-4-silapentane-1-sulfonic acid (DSS) in D_2_O was added to every sample as an internal standard. A ^1^H NMR spectrum of each sample was collected at 25°C on a Bruker Avance 600 spectrometer by using 128 scans and a NOE1D pulse sequence. NMR data were compared to known databases (79), as well as against authentic compounds. The data were processed and analyzed by using CHENOMX (version 8.0) for quantification (45). Data were also analyzed by using Bayesil (80, 81). The average value and the standard error of the mean (SEM) were determined for at least three replicates.

### GFP expression analysis.

Fluorescence levels of translational fusions were determined as previously described (21). Overnight cultures were diluted 1,000× and allowed to grow in M9 minimal medium plus 0.2% Casamino acids and 0.5% glycerol to avoid cell killing by glucose fermentation and to uncouple any effect of acetate accumulation. For *V. cholerae* assays, 50 µM IPTG was added to the medium when appropriate. For *E. coli* assays, 50 µM IPTG and/or 1 mM rhamnose was added to the medium when appropriate. The GFP signal was measured at an optical density at 600 nm (OD_600_) of approximately 0.1 on a BioTek plate reader at 30°C. The number of fluorescence units (FLU) was calculated by dividing the GFP fluorescence signal by the OD_600_. Fifty microliters of mineral oil was added to each well to prevent evaporation in the plate reader. The average value and the SEM were determined for at least three replicates.

### Coculture experiments.

Overnight cultures were diluted to matching ODs and then diluted further to 1,000× for the assay. The assays were done in M9 minimal medium plus 0.2% Casamino acids and 0.5% glucose. Different ratios of LCD (*luxO*^D61E^ Δ*vpsL*) and HCD (Δ*luxO* Δ*vpsL* Δ*lacZ*) or WT (Δ*vpsL* Δ*lacZ*) bacteria were mixed and grown statically for 72 h at 30°C. *V. cholerae* population viability was measured by plating serial dilutions at 24-h intervals on LB–streptomycin–5-bromo-4-chloro-3-indolyl-β-d-galactopyranoside (X-Gal) plates to determine end ratios by using blue versus white colonies plus LB plates to determine total population viability. Limits of detection are indicated in the figure legends. The average value and the SEM were determined for at least three replicates.

## SUPPLEMENTAL MATERIAL

Figure S1  The Δ*vpsL* deletion does not affect strain viability. Strains are grown in M9 minimal medium plus 0.2% Casamino acids with 0.5% glycerol (A), 0.5% glucose (B), or 0.5% GlcNAc (C) as a carbon source. LCD-locked (*luxO*^D61E^ mutant) strains are represented by red lines, and HCD-locked (Δ*luxO* mutant) strains are represented by blue lines. Δ*vpsL* mutant strains are represented by solid squares with dotted lines, while *vpsL*^*+*^ strains are represented by solid circles with solid lines. For panel B, the limit of detection at 48 h is 100 cells/ml of culture, and at 72 h it is 1 cell/ml of culture (open symbol). The values shown are averages of at least three replicates. Error bars denote the SEM. Download Figure S1, TIF file, 0.4 MB

Figure S2  *V. cholerae* strains carrying a WT *luxO* allele (Δ*vpsL* [solid circles] and Δ*aphA* Δ*hapR* Δ*vpsL* [solid squares]) grown in M9 minimal medium plus 0.2% Casamino acids with two different fermentable carbon sources, 0.5% glucose (green) and 0.5% GlcNAc (blue), and a nonfermentable carbon source, 0.5% glycerol (red). The values shown are averages of at least three replicates. Error bars denote the SEM. Download Figure S2, TIF file, 0.2 MB

Figure S3  The glucose sensitivity phenotype is Qrr1-4 mediated. Strains were grown in M9 minimal medium with 0.2% Casamino acids plus 0.5% glucose as a carbon source. LCD-locked (*luxO*^D61E^) strains are represented by red lines, and HCD-locked (Δ*luxO*) strains are represented by blue lines. Δ*aphA* mutant strains are represented by solid circles, Δ*hapR* mutant strains are represented by solid squares, and Δ*aphA* Δ*hapR* mutant strains are represented by solid triangles. For all *luxO*^D61E^ mutant strains, the limit of detection at 48 h is 100 cells/ml of culture and at 72 h it is 2 cells/ml of culture (open symbols). The values shown are averages of at least three replicates. Error bars denote the SEM. Download Figure S3, TIF file, 0.2 MB

Figure S4  Cell viability of QS mutants in different SCM. (A) HCD-locked (Δ*luxO* Δ*aphA* Δ*hapR* Δ*vpsL*) cells were grown in HCD (blue solid circles) and LCD (red solid circles) SCM collected after 72 h of growth. (B) LCD-locked (*luxO*^D61E^ Δ*aphA* Δ*hapR* Δ*vpsL*) cells were grown in HCD (blue solid circles) and LCD (red solid circles) SCM collected after 72 h of growth. The limit of detection at 48 h is 5 cells/ml of culture, and at 72 h it is 1 cell/ml of culture (open symbols). The values shown are averages of at least three replicates. Error bars denote the SEM. Download Figure S4, TIF file, 0.3 MB

Figure S5  Extracellular metabolite profiles of QS mutants grown under fermentative conditions. LCD-locked (*luxO*^D61E^ Δ*aphA* Δ*hapR* Δ*vpsL*) mutants are represented by red triangles, HCD-locked (Δ*luxO* Δ*aphA* Δ*hapR* Δ*vpsL*) mutants are represented by blue squares, a *luxO*^+^ strain (Δ*aphA* Δ*hapR* Δ*vpsL*) is represented by black solid lines and filled circles, and the WT (Δ*vpsL*) is represented by black dotted lines and filled diamonds. Cell viability is shown in panel A. Millimolar concentrations of formate (B), acetate (C), lactate (D), acetoin (E), and 2,3-BD (F) are shown. *P* values (Student’s *t* test) for differences between HCD and LCD mutants: *, *P* < 0.05; **, *P* < 0.01; ***, *P* < 0.001; ****, *P* < 0.0001. The values shown are averages of at least three replicates. Error bars denote the SEM. Download Figure S5, TIF file, 2.1 MB

Figure S6  Organic acids cause cell death. (A) LCD-locked (*luxO*^D61E^ Δ*aphA* Δ*hapR* Δ*vpsL*) cells were grown in fresh M9 minimal medium with 0.5% glucose and 0.2% Casamino acids (red filled circles). HCD-locked (Δ*luxO* Δ*aphA* Δ*hapR* Δ*vpsL*) cells were grown in fresh M9 minimal medium with 0.5% glucose and 0.2% Casamino acids (blue filled squares). HCD-locked (Δ*luxO* Δ*aphA* Δ*hapR* Δ*vpsL*) cells were grown in SCM from an LCD strain grown for 72 h (black asterisks). HCD-locked (Δ*luxO* Δ*aphA* Δ*hapR* Δ*vpsL*) cells were grown in fresh M9 minimal medium with 0.5% glucose and 0.2% Casamino acids plus exogenous organic acids added at 8, 19, 24, and 48 h according to NMR-obtained concentrations of organic acids in the LCD spent medium at those time points. The limit of detection at 48 h is a range of 5 to 100 cells/ml of culture, and at 72 h it is a range of 2 to 100 cells/ml of culture (open symbols). (B) LCD-locked (*luxO*^D61E^ Δ*aphA* Δ*hapR* Δ*vpsL*) cells were grown in SCM collected after 72 h of LCD strain growth (blue filled circles), LCD spent medium buffered with Tris base to pH 7 (black filled triangles), and fresh M9 minimal medium with 0.5% glucose and 0.2% Casamino acids (red filled squares). The limit of detection at 48 h is 100 cells/ml of culture, and at 72 h it is 2 cells/ml of culture (open symbols). The values shown are averages of at least three replicates. Error bars denote the SEM. Download Figure S6, TIF file, 0.3 MB

Figure S7  Qrr1-4 regulate *alsS* translation. Relative GFP fluorescence of different GFP translational fusions in *V. cholerae* HCD-locked (Δ*luxO* Δ*aphA* Δ*hapR* Δ*vpsL*; blue bar) and LCD-locked (*luxO*^D61E^ Δ*aphA* Δ*hapR* Δ*vpsL*; red bar) strains and a *luxO*^+^ strain (Δ*aphA* Δ*hapR* Δ*vpsL*; black bar). Each bar is normalized to the median FLU value of the HCD strain harboring the S1 plasmid. The values shown are averages of at least three replicates. Error bars denote the SEM. *P* values (Student’s *t* test) for differences between HCD and LCD mutants: *, *P* < 0.05; ****, *P* < 0.0001. NS, no statistical significance. Download Figure S7, TIF file, 0.5 MB

Figure S8  Population stability is affected by the presence of the LCD-locked mutants. (A) LCD-locked (*luxO*^D61E^ Δ*vpsL*) cells were cocultured at various percentages in the initial population with WT (Δ*vpsL*) cells under fermentative conditions. The coculture experiments commenced with different set ratios of LCD-locked (*luxO*^D61E^ Δ*vpsL*) QS mutant cells to WT (Δ*vpsL*) cells. Initial ratios consist of 100% WT (purple X), 10% LCD-locked (blue solid squares), 25% LCD-locked (green solid triangles), 50% LCD-locked (orange solid inverted triangles), 75% LCD-locked (yellow solid diamonds), 90% LCD-locked (black solid circles), and 100% LCD-locked (red solid hexagons) cells in M9 minimal medium plus 0.2% Casamino acids and 0.5% glucose. The limit of detection at 72 h is 1 cell/ml of culture (open symbols). (B) The percentage of LCD-locked cells present in the overall culture over time. Shown are ratios from the initial 10% LCD-locked (blue solid squares), 25% LCD-locked (green solid triangles), and 50% LCD-locked (orange solid inverted triangles) cocultures. (C) LCD-locked (*luxO*^D61E^ Δ*vpsL*) QS mutants grown in coculture with HCD-locked (Δ*luxO* Δ*vpsL*) QS mutants and various percentages of LCD-locked cells present in the initial population. The coculture experiments commenced with different ratios of LCD-locked (*luxO*^D61E^ Δ*vpsL*) QS mutant to WT (Δ*vpsL*) cells. Initial ratios consist of 100% HCD-locked (purple X’s), 10% LCD-locked (blue solid squares), 25% LCD-locked (green solid triangles), 50% LCD-locked (orange solid inverted triangles), 75% LCD-locked (yellow solid diamonds), 90% LCD-locked (black solid circles), and 100% LCD-locked (red hexagons) cells in M9 minimal medium plus 0.2% Casamino acids and 0.5% glucose. The limit of detection at 72 h is 1 cell/ml of culture (open symbols). (D) Percentage of LCD-locked QS mutant present in the population over time versus HCD-locked QS mutant cells. Shown are ratios from the initial 10% LCD-locked (blue solid squares), 25% LCD-locked (green solid triangles), and 50% LCD-locked (orange solid inverted triangles) cocultures. The values shown are averages of at least three replicates. Error bars denote the SEM. Download Figure S8, TIF file, 1.1 MB

Table S1  Plasmids and strains used in this study. Table S1, PDF file, 0.1 MB

Table S2  Millimolar concentrations of metabolites found in the initial M9 minimal medium with 0.5% glucose and 0.2% Casamino acids plus SCM of the strains indicated at the 8- and 19-h time points. (A) Values of metabolites in SCM of WT (Δ*vpsL*) cells, HCD-locked (Δ*luxO* Δ*vpsL*) cells, and LCD-locked (*luxO*^D61E^ Δ*vpsL*) cells grown under fermentative conditions were calculated by using Chenomx software and an internal standard of 0.180 mM DSS dissolved in D_2_O. The amino acids arginine, asparagine, cysteine, glutamine, tryptophan, and histidine were not detected in the medium or SCM of any sample. (B) Values of metabolites in the SCM of WT (Δ*vpsL*), HCD-locked (Δ*luxO* Δ*vpsL*), and LCD-locked (*luxO*^D61E^ Δ*vpsL*) cells grown under fermentative conditions were calculated by using Bayesil (80, 81) and an internal standard of 0.180 mM DSS dissolved in D_2_O. The amino acid cysteine was not detected in the medium or SCM of any sample. (C) Values of metabolites in the SCM of WT (Δ*aphA* Δ*hapR* Δ*vpsL*), HCD-locked (Δ*luxO* Δ*aphA* Δ*hapR* Δ*vpsL*), and LCD-locked (*luxO*^D61E^ Δ*aphA* Δ*hapR* Δ*vpsL*) cells grown under fermentative conditions were calculated by using Chenomx software and an internal standard of 0.180 mM DSS dissolved in D_2_O. The amino acids arginine, asparagine, cysteine, glutamine, tryptophan, and histidine were not detected in the medium or SCM of any sample. (D) Values of metabolites in the SCM of WT (Δ*aphA* Δ*hapR* Δ*vpsL*), HCD-locked (Δ*luxO* Δ*aphA* Δ*hapR* Δ*vpsL*), and LCD-locked (*luxO*^D61E^ Δ*aphA* Δ*hapR* Δ*vpsL*) cells grown under fermentative conditions were calculated by using Bayesil (80, 81) and an internal standard of 0.180 mM DSS dissolved in D_2_O. The amino acid cysteine was not detected in the medium or SCM of any sample. The values shown are average millimolar concentrations plus the SEM. N/D indicates that the metabolite was not detected above the limit of detection or the SEM could not be calculated. Values with no SEM are due to only one sample containing the metabolite above the detection limit. Table S2, PDF file, 0.3 MB
