# Supplementary figures and images for: Quorum Sensing Coordinates Cooperative Expression of Pyruvate Metabolism Genes To Maintain a Sustainable Environment for Population Stability
Source: mBio. 2016 Dec 6;7(6):e01863-16. doi: 10.1128/mBio.01863-16 (PMC5142617; doi:10.1128/mBio.01863-16)

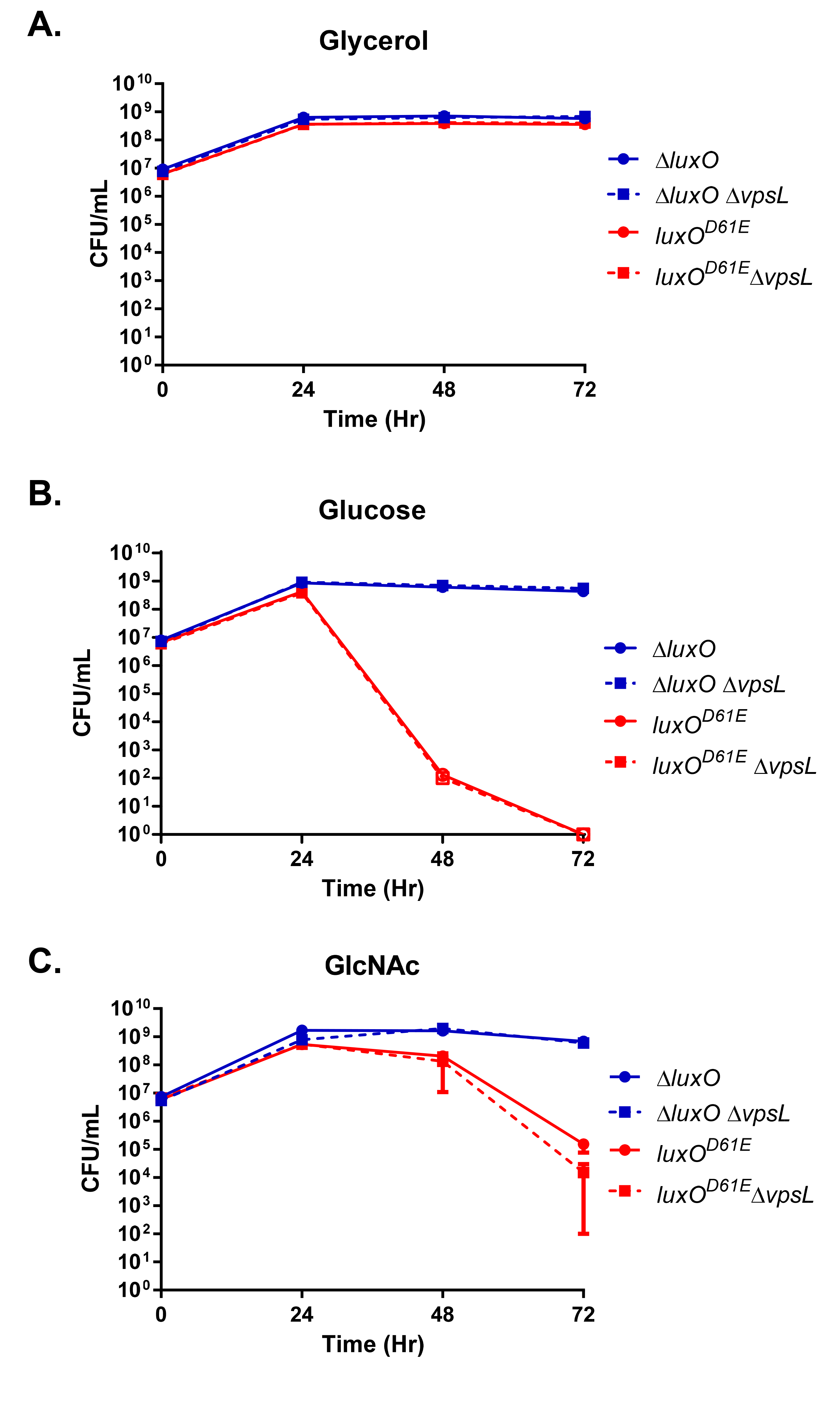

Supplement: Figure S1 — The ΔvpsL deletion does not affect strain viability. Strains are grown in M9 minimal medium plus 0.2% Casamino acids with 0.5% glycerol (A), 0.5% glucose (B), or 0.5% GlcNAc (C) as a carbon source. LCD-locked (luxOD61E mutant) strains are represented by red lines, and HCD-locked (ΔluxO mutant) strains are represented by blue lines. ΔvpsL mutant strains are represented by solid squares with dotted lines, while vpsL+ strains are represented by solid circles with solid lines. For panel B, the limit of detection at 48 h is 100 cells/ml of culture, and at 72 h it is 1 cell/ml of culture (open symbol). The values shown are averages of at least three replicates. Error bars denote the SEM. Download [file mbo006163094sf1.tif]

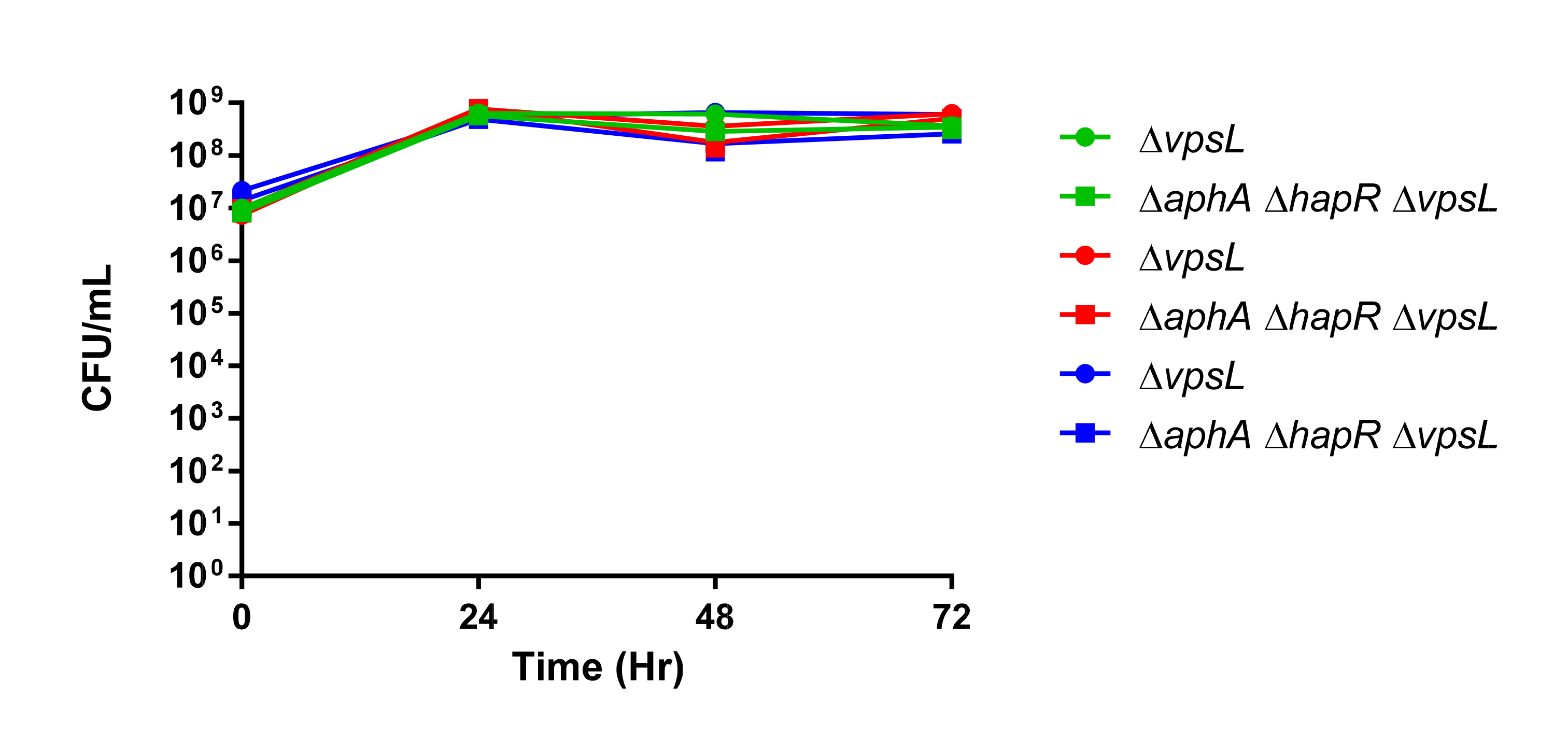

Supplement: Figure S2 — V. cholerae strains carrying a WT luxO allele (ΔvpsL [solid circles] and ΔaphA ΔhapR ΔvpsL [solid squares]) grown in M9 minimal medium plus 0.2% Casamino acids with two different fermentable carbon sources, 0.5% glucose (green) and 0.5% GlcNAc (blue), and a nonfermentable carbon source, 0.5% glycerol (red). The values shown are averages of at least three replicates. Error bars denote the SEM. Download [file mbo006163094sf2.tif]

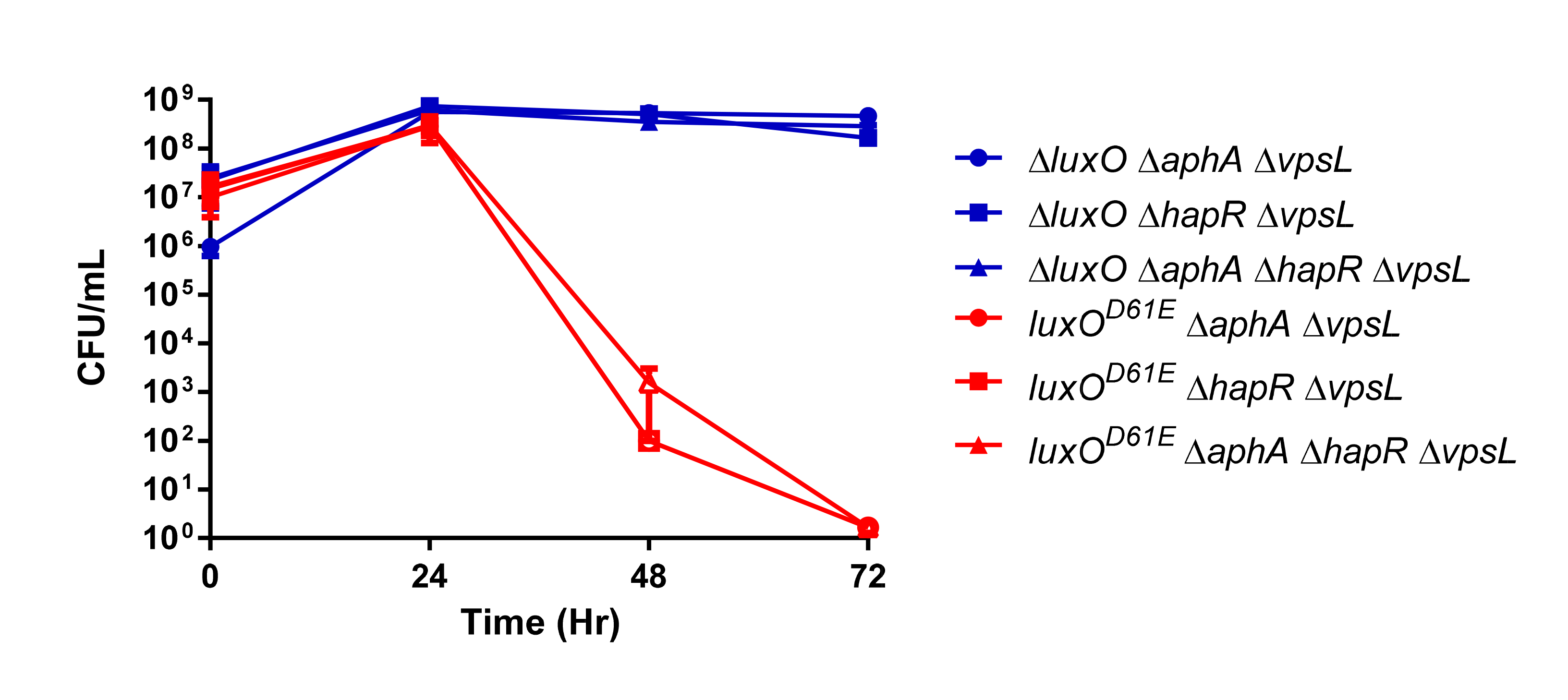

Supplement: Figure S3 — The glucose sensitivity phenotype is Qrr1-4 mediated. Strains were grown in M9 minimal medium with 0.2% Casamino acids plus 0.5% glucose as a carbon source. LCD-locked (luxOD61E) strains are represented by red lines, and HCD-locked (ΔluxO) strains are represented by blue lines. ΔaphA mutant strains are represented by solid circles, ΔhapR mutant strains are represented by solid squares, and ΔaphA ΔhapR mutant strains are represented by solid triangles. For all luxOD61E mutant strains, the limit of detection at 48 h is 100 cells/ml of culture and at 72 h it is 2 cells/ml of culture (open symbols). The values shown are averages of at least three replicates. Error bars denote the SEM. Download [file mbo006163094sf3.tif]

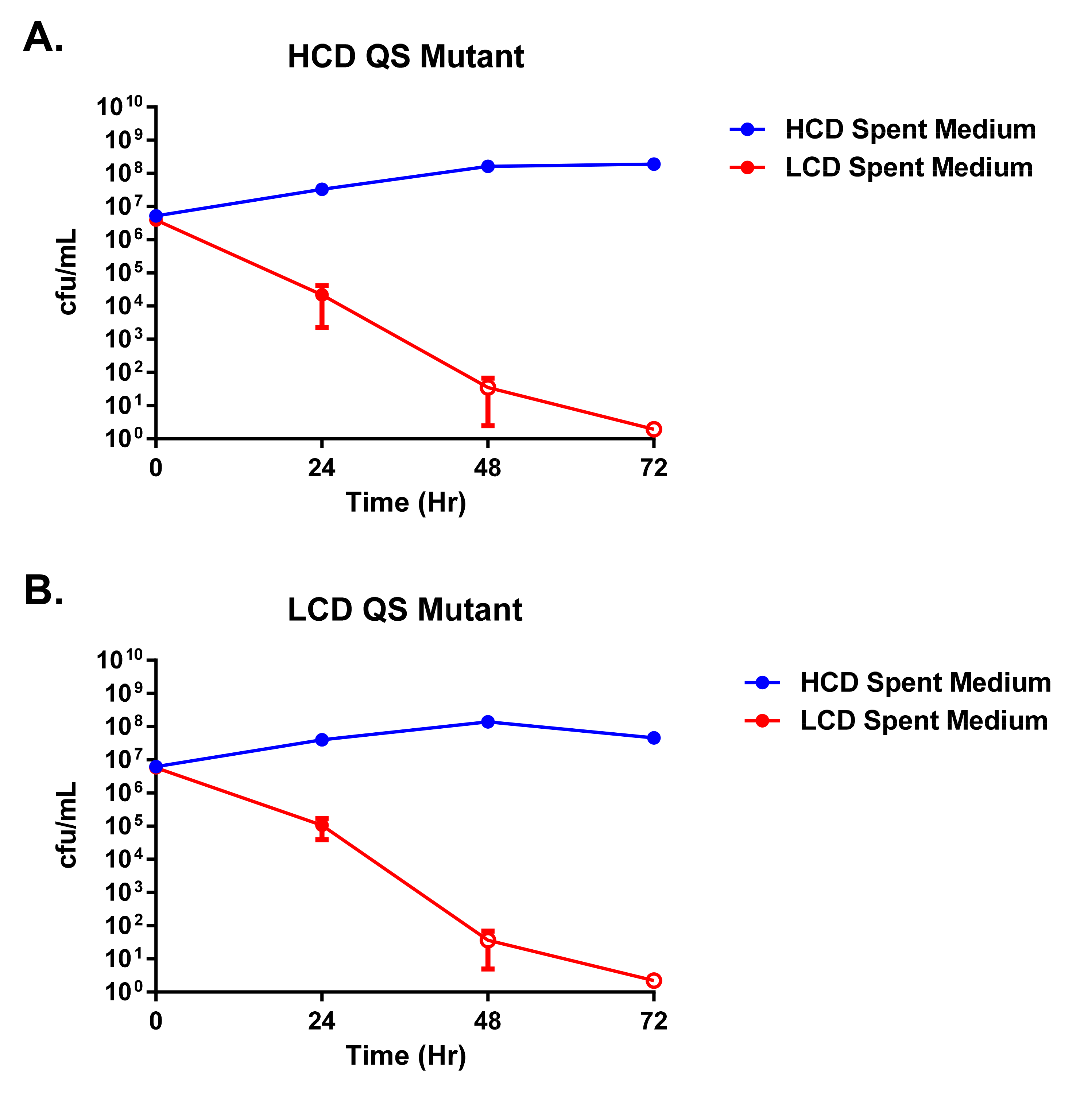

Supplement: Figure S4 — Cell viability of QS mutants in different SCM. (A) HCD-locked (ΔluxO ΔaphA ΔhapR ΔvpsL) cells were grown in HCD (blue solid circles) and LCD (red solid circles) SCM collected after 72 h of growth. (B) LCD-locked (luxOD61E ΔaphA ΔhapR ΔvpsL) cells were grown in HCD (blue solid circles) and LCD (red solid circles) SCM collected after 72 h of growth. The limit of detection at 48 h is 5 cells/ml of culture, and at 72 h it is 1 cell/ml of culture (open symbols). The values shown are averages of at least three replicates. Error bars denote the SEM. Download [file mbo006163094sf4.tif]

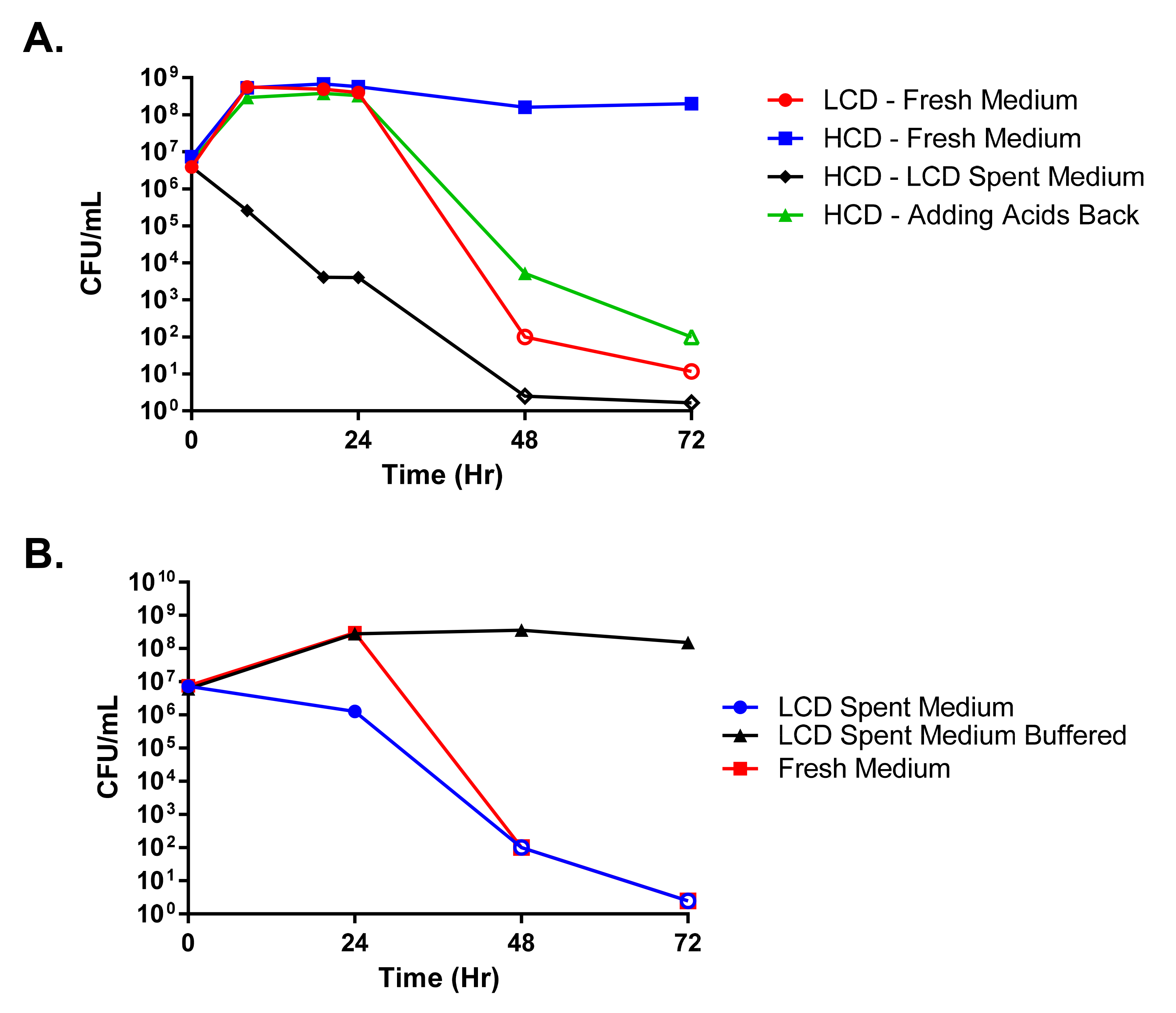

Supplement: Figure S6 — Organic acids cause cell death. (A) LCD-locked (luxOD61E ΔaphA ΔhapR ΔvpsL) cells were grown in fresh M9 minimal medium with 0.5% glucose and 0.2% Casamino acids (red filled circles). HCD-locked (ΔluxO ΔaphA ΔhapR ΔvpsL) cells were grown in fresh M9 minimal medium with 0.5% glucose and 0.2% Casamino acids (blue filled squares). HCD-locked (ΔluxO ΔaphA ΔhapR ΔvpsL) cells were grown in SCM from an LCD strain grown for 72 h (black asterisks). HCD-locked (ΔluxO ΔaphA ΔhapR ΔvpsL) cells were grown in fresh M9 minimal medium with 0.5% glucose and 0.2% Casamino acids plus exogenous organic acids added at 8, 19, 24, and 48 h according to NMR-obtained concentrations of organic acids in the LCD spent medium at those time points. The limit of detection at 48 h is a range of 5 to 100 cells/ml of culture, and at 72 h it is a range of 2 to 100 cells/ml of culture (open symbols). (B) LCD-locked (luxOD61E ΔaphA ΔhapR ΔvpsL) cells were grown in SCM collected after 72 h of LCD strain growth (blue filled circles), LCD spent medium buffered with Tris base to pH 7 (black filled triangles), and fresh M9 minimal medium with 0.5% glucose and 0.2% Casamino acids (red filled squares). The limit of detection at 48 h is 100 cells/ml of culture, and at 72 h it is 2 cells/ml of culture (open symbols). The values shown are averages of at least three replicates. Error bars denote the SEM. Download [file mbo006163094sf6.tif]

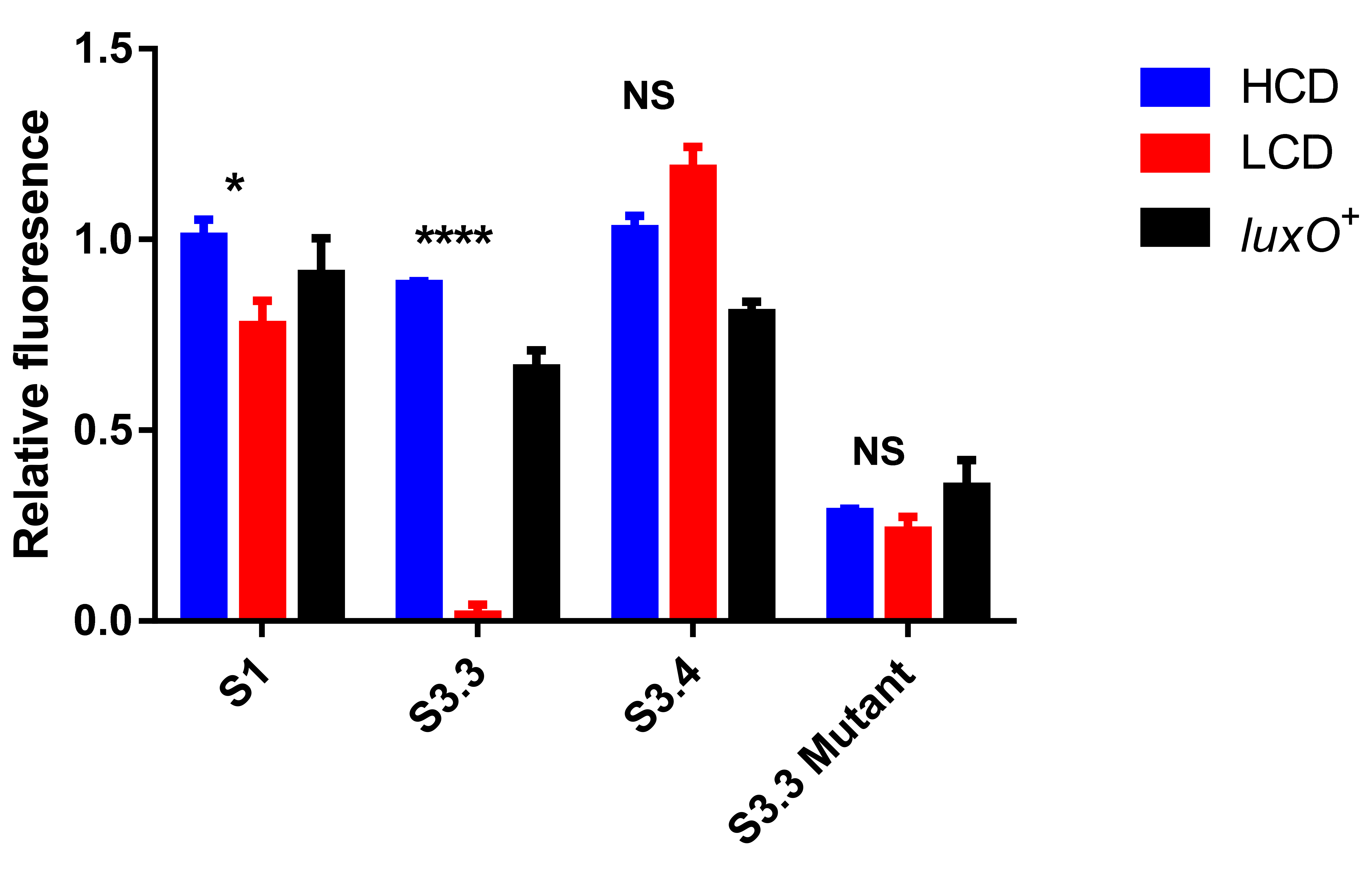

Supplement: Figure S7 — Qrr1-4 regulate alsS translation. Relative GFP fluorescence of different GFP translational fusions in V. cholerae HCD-locked (ΔluxO ΔaphA ΔhapR ΔvpsL; blue bar) and LCD-locked (luxOD61E ΔaphA ΔhapR ΔvpsL; red bar) strains and a luxO+ strain (ΔaphA ΔhapR ΔvpsL; black bar). Each bar is normalized to the median FLU value of the HCD strain harboring the S1 plasmid. The values shown are averages of at least three replicates. Error bars denote the SEM. P values (Student’s t test) for differences between HCD and LCD mutants: *, P < 0.05; ****, P < 0.0001. NS, no statistical significance. Download [file mbo006163094sf7.tif]

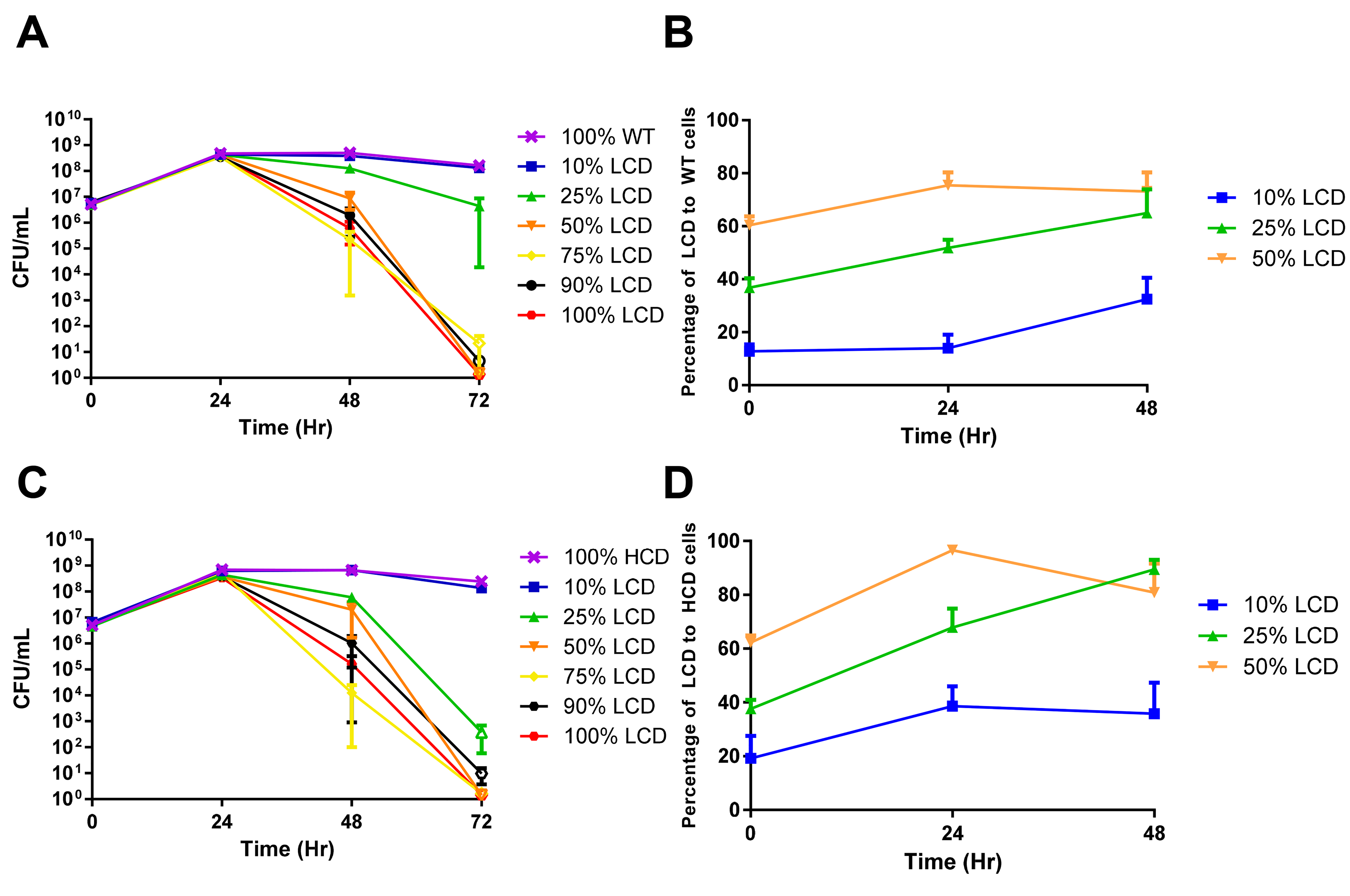

Supplement: Figure S8 — Population stability is affected by the presence of the LCD-locked mutants. (A) LCD-locked (luxOD61E ΔvpsL) cells were cocultured at various percentages in the initial population with WT (ΔvpsL) cells under fermentative conditions. The coculture experiments commenced with different set ratios of LCD-locked (luxOD61E ΔvpsL) QS mutant cells to WT (ΔvpsL) cells. Initial ratios consist of 100% WT (purple X), 10% LCD-locked (blue solid squares), 25% LCD-locked (green solid triangles), 50% LCD-locked (orange solid inverted triangles), 75% LCD-locked (yellow solid diamonds), 90% LCD-locked (black solid circles), and 100% LCD-locked (red solid hexagons) cells in M9 minimal medium plus 0.2% Casamino acids and 0.5% glucose. The limit of detection at 72 h is 1 cell/ml of culture (open symbols). (B) The percentage of LCD-locked cells present in the overall culture over time. Shown are ratios from the initial 10% LCD-locked (blue solid squares), 25% LCD-locked (green solid triangles), and 50% LCD-locked (orange solid inverted triangles) cocultures. (C) LCD-locked (luxOD61E ΔvpsL) QS mutants grown in coculture with HCD-locked (ΔluxO ΔvpsL) QS mutants and various percentages of LCD-locked cells present in the initial population. The coculture experiments commenced with different ratios of LCD-locked (luxOD61E ΔvpsL) QS mutant to WT (ΔvpsL) cells. Initial ratios consist of 100% HCD-locked (purple X’s), 10% LCD-locked (blue solid squares), 25% LCD-locked (green solid triangles), 50% LCD-locked (orange solid inverted triangles), 75% LCD-locked (yellow solid diamonds), 90% LCD-locked (black solid circles), and 100% LCD-locked (red hexagons) cells in M9 minimal medium plus 0.2% Casamino acids and 0.5% glucose. The limit of detection at 72 h is 1 cell/ml of culture (open symbols). (D) Percentage of LCD-locked QS mutant present in the population over time versus HCD-locked QS mutant cells. Shown are ratios from the initial 10% LCD-locked (blue solid squares), 25% LCD-locked (green solid [file mbo006163094sf8.tif]
