# Supplementary material for: Quorum Sensing Coordinates Cooperative Expression of Pyruvate Metabolism Genes To Maintain a Sustainable Environment for Population Stability
Source: mBio. 2016 Dec 6;7(6):e01863-16. doi: 10.1128/mBio.01863-16 (PMC5142617; doi:10.1128/mBio.01863-16)
Supplement: Table S1 — Plasmids and strains used in this study. [file mbo006163094st1.pdf]

SUPPORTING INFORMATION

Table S1. Bacterial strains used in this study.

Strain Number	Relevant Genotype	Antibiotic Resistance(s)	Reference
V. cholerae strains			
WN0001	C6706 str2	SmR	(1)
WN0072	Δ*luxO*	SmR	(2)
WN0778	*luxO*^{D61E}	SmR	(2)
WN0862	Δ*vpsL*	SmR	(3)
WN0863	*luxO*^{D61E} Δ*vpsL*	SmR	(3)
WN0865	Δ*luxO* Δ*vpsL*	SmR	(3)
WN0868	Δ*luxO* Δ*hapR* Δ*vpsL*	SmR	This study
WN2066	Δ*aphA* Δ*hapR* Δ*vpsL*	SmR	This study
WN2079	*luxO*^{D61E} Δ*hapR* Δ*vpsL*	SmR	This study
WN2089	*luxO*^{D61E} Δ*aphA* Δ*vpsL*	SmR	This study
WN2090	Δ*luxO* Δ*aphA* Δ*vpsL*	SmR	This study
WN2091	Δ*luxO* Δ*aphA* Δ*hapR* Δ*vpsL*	SmR	This study
WN2092	*luxO*^{D61E}Δ*aphA* Δ*hapR* Δ*vpsL*	SmR	This study
WN3126	Δ*lacZ* Δ*vpsL*	SmR	This study
WN3192	*luxO*^{D61E} Δ*aphA* Δ*hapR* Δ*vpsL* pFED342	KanR	This study
WN3751	Δ*luxO* Δ*aphA* Δ*hapR* Δ*vpsL* pFED342	KanR	This study
WN3779	Δ*luxO* Δ*lacZ* Δ*vpsL*	SmR	This study
WN4102	*luxO*^{D61E} Δ*aphA* Δ*hapR* Δ*vpsL* pEVS143-*alsDSO*	KanR	This study
WN4171	*luxO*^{D61E} Δ*aphA* Δ*hapR* Δ*vpsL* pEVS143-*alsD*	KanR	This study
WN4172	*luxO*^{D61E} Δ*aphA* Δ*hapR* Δ*vpsL* pEVS143-*alsS*	KanR	This study
WN4173	*luxO*^{D61E} Δ*aphA* Δ*hapR* Δ*vpsL* pEVS143-*alsO*	KanR	This study
WN4236	*luxO*^{D61E} Δ*aphA* Δ*hapR* Δ*vpsL* pEVS143-*alsR*	KanR	This study
WN4405	Δ*luxO* Δ*aphA* Δ*hapR* Δ*vpsL* pEVS141-P_{tac}-*qrr4*	KanR	This study
WN4446	Δ*luxO* Δ*aphA* Δ*hapR* Δ*vpsL* Δ*alsDSO*	SmR	This study
WN4504	Δ*luxO* Δ*aphA* Δ*hapR* Δ*vpsL* pEVS143-*alsS1-gfp*^{LVA}	KanR	This study
WN4509	*luxO*^{D61E} Δ*aphA* Δ*hapR* Δ*vpsL* pEVS143-*alsS1-gfp*^{LVA}	KanR	This study
WN4938	Δ*luxO* Δ*aphA* Δ*hapR* Δ*vpsL* pEVS143-*alsS3.3-gfp*^{LVA}	KanR	This study
WN4940	*luxO*^{D61E} Δ*aphA* Δ*hapR* Δ*vpsL* pEVS143-*alsS3.3-gfp*^{LVA}	KanR	This study
WN4942	Δ*luxO* Δ*aphA* Δ*hapR* Δ*vpsL* pEVS143-*alsS3.4-gfp*^{LVA}	KanR	This study
WN4944	*luxO*^{D61E} Δ*aphA* Δ*hapR* Δ*vpsL* pEVS143-*alsS3.4-gfp*^{LVA}	KanR	This study
WN5109	Δ*luxO* Δ*aphA* Δ*hapR* Δ*vpsL* pEVS143-*alsS3.3*Mutant-*gfp*^{LVA}	KanR	This study
WN5110	*luxO*^{D61E} Δ*aphA* Δ*hapR* Δ*vpsL* pEVS143-*alsS3.3*Mutant-*gfp*^{LVA}	KanR	This study
WN5221	Δ*aphA* Δ*hapR* Δ*vpsL* pEVS143-*alsS1-gfp*^{LVA}	KanR	This study
WN5224	Δ*aphA* Δ*hapR* Δ*vpsL* pEVS143-*alsS3.3-gfp*^{LVA}	KanR	This study
WN5227	Δ*aphA* Δ*hapR* Δ*vpsL* pEVS143-*alsS3.4-gfp*^{LVA}	KanR	This study
WN5230	Δ*aphA* Δ*hapR* Δ*vpsL* pEVS143-*alsS3.3*Mutant-*gfp*^{LVA}	KanR	This study

E. coli strains			
WN0006	S17-1λ*pir* pKAS32	AmpR	(4)
WN0092	S17-1λ*pir* pEVS143	KanR	(5)
WN0479	S17-1λ*pir* pFED342	KanR	(6)
WN1438	S17-1λ*pir* pKAS32-Δ*lacZ*	AmpR	(7)
WN3015	S17-1λ*pir* pEVS141-P_{tac}-*qrr4*	KanR	(8)
WN4098	S17-1λ*pir* pEVS143-*alsDSO*	KanR	This study
WN4143	S17-1λ*pir* pEVS143-*alsD*	KanR	This study
WN4145	S17-1λ*pir* pEVS143-*alsS*	KanR	This study
WN4147	S17-1λ*pir* pEVS143-*alsO*	KanR	This study
WN4210	S17-1λ*pir* pEVS143-*alsR*	KanR	This study
WN4379	S17-1λ*pir* pKAS32-Δ*alsDSO*	AmpR	This study
WN4493	S17-1λ*pir* pEVS143-*alsS1*-gfp^{LVA}	KanR	This study
WN4522	S17-1λ*pir* pEVS143-gfp^{LVA}	KanR	This study
WN4531	S17-1λ*pir* pEVS143-*alsS1*-gfp^{LVA} and pRHA109	KanR AmpR	This study
WN4534	S17-1λ*pir* pEVS143-gfp^{LVA} and pRHA109	KanR AmpR	This study
WN4559	S17-1λ*pir* pRHA109	AmpR	(9)
WN4689	S17-1λ*pir* pRHA109-*qrr4*	AmpR	This study
WN4694	S17-1λ*pir* pEVS143-*alsS1*-gfp^{LVA} and pRHA109 *qrr4*	KanR AmpR	This study
WN4699	S17-1λ*pir* pEVS143-gfp^{LVA} and pRHA109 *qrr4*	KanR AmpR	This study
WN4776	S17-1λ*pir* pEVS143-*alsS3.3*-gfp^{LVA}	KanR	This study
WN4792	S17-1λ*pir* pEVS143-*alsS3.3*-gfp^{LVA} and pRHA109	KanR AmpR	This study
WN4796	S17-1λ*pir* pEVS143-*alsS3.3*-gfp^{LVA} and pRHA109 *qrr4*	KanR AmpR	This study
WN4819	S17-1λ*pir* pEVS143-*alsS3.4*-gfp^{LVA}	KanR	This study
WN4830	S17-1λ*pir* pEVS143-*alsS3.4*-gfp^{LVA} and pRHA109	KanR AmpR	This study
WN4834	S17-1λ*pir* pEVS143-*alsS3.4*-gfp^{LVA} and pRHA109 *qrr4*	KanR AmpR	This study
WN5085	S17-1λ*pir* pEVS143-*alsS3.3*Mutant-gfp^{LVA}	KanR	This study

References

1. Thelin KH & Taylor RK (1996) Toxin-coregulated pilus, but not mannose-sensitive hemagglutinin, is required for colonization by *Vibrio cholerae* O1 El Tor biotype and O139 strains. *Infect Immun* 64(7):2853-2856.
2. Hammer BK & Bassler BL (2009) Distinct sensory pathways in *Vibrio cholerae* El Tor and classical biotypes modulate cyclic dimeric GMP levels to control biofilm formation. *J Bacteriol* 191(1):169-177.
3. Waters CM, Lu W, Rabinowitz JD, & Bassler BL (2008) Quorum sensing controls biofilm formation in *Vibrio cholerae* through modulation of cyclic di-GMP levels and repression of vpsT. *J Bacteriol* 190(7):2527-2536.
4. Skorupski K & Taylor RK (1996) Positive selection vectors for allelic exchange. *Gene* 169(1):47-52.
5. Bose JL, Rosenberg CS, & Stabb EV (2008) Effects of *luxCDABEG* induction in *Vibrio fischeri*: enhancement of symbiotic colonization and conditional attenuation of growth in culture. *Arch Microbiol* 190(2):169-183.
6. Svenningsen SL, Waters CM, & Bassler BL (2008) A negative feedback loop involving small RNAs accelerates *Vibrio cholerae*'s transition out of quorum-sensing mode. *Genes Dev* 22(2):226-238.

7. Miller MB, Skorupski K, Lenz DH, Taylor RK, & Bassler BL (2002) Parallel quorum sensing systems converge to regulate virulence in *Vibrio cholerae*. *Cell* 110(3):303-314 .
8. Svenningsen SL, Tu KC, & Bassler BL (2009) Gene dosage compensation calibrates four regulatory RNAs to control *Vibrio cholerae* quorum sensing. *EMBO J* 28(4):429-439 .
9. Giacalone MJ*, et al.* (2006) Toxic protein expression in *Escherichia coli* using a rhamnose-based tightly regulated and tunable promoter system. *Biotechniques* 40(3):355-364.
