# Supplementary material for: Quorum Sensing Coordinates Cooperative Expression of Pyruvate Metabolism Genes To Maintain a Sustainable Environment for Population Stability
Source: mBio. 2016 Dec 6;7(6):e01863-16. doi: 10.1128/mBio.01863-16 (PMC5142617; doi:10.1128/mBio.01863-16)
Supplement: Table S2 — Millimolar concentrations of metabolites found in the initial M9 minimal medium with 0.5% glucose and 0.2% Casamino acids plus SCM of the strains indicated at the 8- and 19-h time points. (A) Values of metabolites in SCM of WT (ΔvpsL) cells, HCD-locked (ΔluxO ΔvpsL) cells, and LCD-locked (luxOD61E ΔvpsL) cells grown under fermentative conditions were calculated by using Chenomx software and an internal standard of 0.180 mM DSS dissolved in D2O. The amino acids arginine, asparagine, cysteine, glutamine, tryptophan, and histidine were not detected in the medium or SCM of any sample. (B) Values of metabolites in the SCM of WT (ΔvpsL), HCD-locked (ΔluxO ΔvpsL), and LCD-locked (luxOD61E ΔvpsL) cells grown under fermentative conditions were calculated by using Bayesil (80, 81) and an internal standard of 0.180 mM DSS dissolved in D2O. The amino acid cysteine was not detected in the medium or SCM of any sample. (C) Values of metabolites in the SCM of WT (ΔaphA ΔhapR ΔvpsL), HCD-locked (ΔluxO ΔaphA ΔhapR ΔvpsL), and LCD-locked (luxOD61E ΔaphA ΔhapR ΔvpsL) cells grown under fermentative conditions were calculated by using Chenomx software and an internal standard of 0.180 mM DSS dissolved in D2O. The amino acids arginine, asparagine, cysteine, glutamine, tryptophan, and histidine were not detected in the medium or SCM of any sample. (D) Values of metabolites in the SCM of WT (ΔaphA ΔhapR ΔvpsL), HCD-locked (ΔluxO ΔaphA ΔhapR ΔvpsL), and LCD-locked (luxOD61E ΔaphA ΔhapR ΔvpsL) cells grown under fermentative conditions were calculated by using Bayesil (80, 81) and an internal standard of 0.180 mM DSS dissolved in D2O. The amino acid cysteine was not detected in the medium or SCM of any sample. The values shown are average millimolar concentrations plus the SEM. N/D indicates that the metabolite was not detected above the limit of detection or the SEM could not be calculated. Values with no SEM are due to only one sample containing the metabolite above the detection limit. [file mbo006163094st2.pdf]

Table S2A - Chenomx Analysis of Δ*vpsL* strains

	Media (0 Hrs)	WT 8 Hrs		WT 19 Hrs		HCD 8 Hrs		HCD 19 Hrs		LCD 8 Hrs		LCD 19 Hrs		HCD/WT 8 Hrs	HCD/WT 19 Hrs	LCD/WT 8 Hrs	LCD/WT 19 Hrs	HCD/LCD 8 Hrs	HCD/LCD 19 Hrs	LCD/HCD 8 Hrs	LCD/HCD 19 Hrs
	[mM]	[mM]	+/-	[mM]	+/-	[mM]	+/-	[mM]	+/-	[mM]	+/-	[mM]	+/-	Fold Change	Fold Change	Fold Change	Fold Change	Fold Change	Fold Change	Fold Change	Fold Change
2,3 Butanediol	N/D	0.684	0.268	3.617	1.085	0.32	0.01	2.18	0.09	0.09	0.01	0.70	0.04	0.46	0.60	0.13	0.19	3.70	3.09	0.27	0.32
Acetate	0.10	3.892	0.352	3.131	0.492	2.89	0.04	3.72	0.24	5.14	0.13	7.75	0.21	0.74	1.19	1.32	2.47	0.56	0.48	1.78	2.08
Acetoin	N/D	0.516	0.216	0.755	0.295	0.30	0.03	0.93	0.04	0.05	0.00	0.26	0.02	0.58	1.24	0.09	0.35	6.57	3.57	0.15	0.28
Alanine	0.36	0.262	0.019	0.330	0.006	0.27	0.01	0.29	0.02	0.30	0.00	0.35	0.01	1.03	0.88	1.13	1.07	0.91	0.83	1.10	1.21
Aspartate	0.58	0.065	0.017	0.039	0.009	0.04	0.00	0.04	0.01	0.04	0.00	0.04	0.00	0.63	1.15	0.60	1.03	1.05	1.12	0.95	0.89
Ethanol	N/D	3.843	0.660	7.622	0.998	2.27	0.05	4.86	0.24	3.41	0.09	5.07	0.12	0.59	0.64	0.89	0.67	0.66	0.96	1.51	1.04
Formate	0.02	7.784	0.795	12.030	0.412	4.72	0.14	8.24	0.47	7.89	0.21	10.62	0.29	0.61	0.68	1.01	0.88	0.60	0.78	1.67	1.29
Glucose	27.74	14.518	0.497	4.129	3.184	17.69	0.20	8.23	0.48	15.85	0.20	10.50	0.24	1.22	1.99	1.09	2.54	1.12	0.78	0.90	1.28
Glutamate	1.01	0.486	0.136	0.544	0.168	0.72	0.02	0.71	0.03	0.76	0.00	0.68	0.04	1.49	1.31	1.57	1.26	0.95	1.04	1.05	0.96
Glycine	0.22	0.111	0.003	0.146	0.014	0.10	0.00	0.10	0.01	0.10	0.01	0.13	0.01	0.91	0.70	0.91	0.89	1.00	0.79	1.00	1.26
Isoleucine	0.21	0.193	0.023	0.201	0.016	0.21	0.02	0.16	0.01	0.18	0.00	0.16	0.01	1.08	0.78	0.91	0.81	1.19	0.97	0.84	1.03
Lactate	0.01	0.171	0.036	0.385	0.062	0.03	0.00	0.16	0.01	0.64	0.01	3.63	0.09	0.18	0.43	3.75	9.42	0.05	0.05	21.38	22.08
Leucine	0.53	0.419	0.046	0.452	0.027	0.42	0.00	0.37	0.01	0.40	0.01	0.43	0.01	1.01	0.81	0.97	0.94	1.04	0.86	0.96	1.17
Lysine	0.04	0.163	0.069	0.246	0.110	0.23	0.00	0.20	0.03	0.25	0.03	0.07	0.01	1.40	0.80	1.52	0.28	0.92	2.83	1.09	0.35
Methionine	0.11	0.137	0.017	0.135	0.019	0.12	0.00	0.11	0.00	0.11	0.01	0.11	0.00	0.87	0.79	0.77	0.81	1.13	0.98	0.88	1.02
Phenylalanine	0.14	0.139	0.025	0.152	0.011	0.12	0.00	0.12	0.01	0.12	0.00	0.12	0.00	0.86	0.79	0.87	0.77	0.99	1.03	1.01	0.97
Proline	0.62	0.336	0.057	0.282	0.031	0.41	0.08	0.46	0.02	0.40	0.03	0.32	0.03	1.23	1.65	1.19	1.15	1.03	1.43	0.97	0.70
Pyroglutamate	0.71	0.415	0.033	0.406	0.043	0.54	0.02	0.54	0.01	0.44	0.02	0.41	0.06	1.29	1.34	1.05	1.01	1.22	1.33	0.82	0.75
Pyruvate	N/D	0.111	0.031	0.057	0.006	0.12	0.00	0.05	0.00	1.11	0.01	0.88	0.04	1.05	0.89	9.98	15.36	0.11	0.06	9.51	17.28
Serine	0.05	N/D	N/D	N/D	N/D	N/D	N/D	N/D	N/D	N/D	N/D	N/D	N/D	N/D	N/D	N/D	N/D	N/D	N/D	N/D	N/D
Succinate	N/D	0.764	0.172	1.991	0.461	0.53	0.01	1.47	0.05	0.53	0.02	1.06	0.03	0.70	0.74	0.69	0.53	1.01	1.39	0.99	0.72
Threonine	0.23	0.078	0.027	0.078	0.027	0.18	0.00	0.03	0.03	0.07	0.02	0.10	0.00	2.28	0.44	0.91	1.31	2.51	0.33	0.40	3.00
Tyrosine	0.08	0.083	0.015	0.091	0.007	0.07	0.00	0.08	0.00	0.07	0.00	0.07	0.00	0.82	0.83	0.82	0.80	1.00	1.04	1.00	0.96
Valine	0.36	0.324	0.032	0.338	0.019	0.33	0.01	0.27	0.01	0.30	0.01	0.26	0.04	1.02	0.79	0.91	0.78	1.12	1.01	0.90	0.99

Table S2B - Bayesil Analysis of Δ*vpsL* strains

	Media (0 Hrs)	WT 8 Hrs		WT 19 Hrs		HCD 8 Hrs		HCD 19 Hrs		LCD 8 Hrs		LCD 19 Hrs		HCD/WT 8 Hrs	HCD/WT 19 Hrs	LCD/WT 8 Hrs	LCD/WT 19 Hrs	HCD/LCD 8 Hrs	HCD/LCD 19 Hrs	LCD/HCD 8 Hrs	LCD/HCD 19 Hrs
	[mM]	[mM]	+/-	[mM]	+/-	[mM]	+/-	[mM]	+/-	[mM]	+/-	[mM]	+/-	Fold Change	Fold Change	Fold Change	Fold Change	Fold Change	Fold Change	Fold Change	Fold Change
Acetate	0.02	2.71	0.24	2.12	0.31	0.89	0.02	1.15	0.07	1.60	0.03	2.43	0.06	0.33	0.54	0.59	1.14	0.55	0.48	1.80	2.10
Acetoacetate	N/D	N/D	N/D	N/D	N/D	N/D	N/D	N/D	N/D	N/D	N/D	N/D	N/D	N/D	N/D	N/D	N/D	N/D	N/D	N/D	N/D
Acetone	N/D	0.28	0.12	0.42	0.16	0.12	0.00	0.49	0.02	0.02	0.00	0.14	0.01	0.41	1.15	0.08	0.32	5.30	3.60	0.19	0.28
Alanine	0.14	0.17	0.01	0.23	N/D	0.11	0.00	0.13	0.01	0.14	0.00	0.17	0.00	0.66	0.57	0.83	0.75	0.79	0.76	1.26	1.32
Arginine	0.05	0.03	0.03	0.04	0.04	N/D	N/D	0.03	0.02	0.02	0.02	N/D	N/D	N/D	0.85	0.84	N/D	N/D	N/D	N/D	N/D
Asparagine	0.05	0.02	0.01	0.02	0.01	0.02	0.01	0.02	0.01	0.02	0.01	0.02	0.00	1.12	0.83	0.86	0.74	1.30	1.12	0.77	0.89
Aspartate	0.60	0.03	0.02	0.02	0.01	0.04	0.01	0.01	0.00	0.02	0.00	0.03	0.00	1.25	0.41	0.71	1.51	1.77	0.27	0.57	3.67
Ethanol	0.03	4.29	0.65	8.56	1.17	2.16	0.06	4.88	0.28	3.20	0.06	5.16	0.10	0.50	0.57	0.75	0.60	0.67	0.95	1.49	1.06
Formate	0.02	8.36	0.79	12.48	0.68	4.53	0.11	7.84	0.45	7.52	0.16	10.11	0.30	0.54	0.63	0.90	0.81	0.60	0.78	1.66	1.29
Glucose	25.82	13.69	0.51	3.89	3.29	14.98	0.35	6.33	0.60	13.43	0.03	8.64	0.09	1.09	1.63	0.98	2.22	1.12	0.73	0.90	1.36
Glutamic acid	1.82	0.91	0.10	0.99	0.02	1.30	0.02	1.45	0.17	1.33	0.01	1.12	0.08	1.43	1.46	1.45	1.12	0.98	1.30	1.02	0.77
Glutamine	0.31	0.07	0.01	N/D	N/D	0.01	0.01	0.04	0.02	0.07	0.02	0.36	0.08	0.09	N/D	0.97	N/D	0.09	0.10	11.16	9.64
Glycine	0.67	0.03	0.02	0.06	0.04	0.06	0.02	0.03	0.02	0.11	0.01	0.03	0.02	1.73	0.57	3.24	0.54	0.53	1.06	1.87	0.94
Histidine	0.24	N/D	N/D	N/D	N/D	0.07	0.04	0.07	0.04	0.08	0.02	0.06	0.02	N/D	N/D	N/D	N/D	0.83	1.15	1.20	0.87
Isoleucine	0.30	0.24	0.03	0.20	0.04	0.19	0.01	0.17	0.01	0.20	0.00	0.17	0.01	0.77	0.86	0.82	0.85	0.95	1.01	1.05	0.99
Lactate	0.03	0.16	0.04	0.34	0.08	0.09	0.01	0.21	0.01	0.91	0.05	4.55	0.10	0.59	0.61	5.82	13.42	0.10	0.05	9.91	21.98
Leucine	0.61	0.45	0.05	0.43	0.04	0.38	0.01	0.36	0.03	0.40	0.00	0.36	0.01	0.84	0.84	0.89	0.84	0.94	1.00	1.06	1.00
Lysine	0.28	0.28	0.03	0.44	0.03	0.26	0.01	0.27	0.00	0.22	0.04	0.35	0.01	0.92	0.63	0.79	0.81	1.16	0.78	0.86	1.29
Malonate	0.14	0.02	0.01	0.01	N/D	0.02	0.00	0.01	0.00	0.02	0.00	0.01	0.00	0.66	1.04	0.66	1.31	1.00	0.79	1.00	1.27
Methionine	0.10	0.11	0.01	0.12	N/D	0.10	0.00	0.09	0.00	0.09	0.00	0.10	0.00	0.93	0.80	0.84	0.86	1.10	0.94	0.91	1.07
Phenylalanine	0.16	0.13	0.01	0.15	N/D	0.12	0.00	0.12	0.01	0.11	0.00	0.12	0.00	0.88	0.80	0.84	0.82	1.05	0.98	0.95	1.02
Proline	0.63	0.36	0.02	0.27	0.07	0.39	0.02	0.28	0.01	0.12	0.01	0.23	0.06	1.08	1.02	0.33	0.85	3.24	1.20	0.31	0.83
Pyruvic acid	0.02	0.04	N/D	0.04	0.01	0.12	0.00	0.03	0.01	1.03	0.01	0.84	0.10	2.66	0.73	23.67	19.40	0.11	0.04	8.91	26.42
Serine	1.51	0.03	0.03	0.07	0.04	N/D	N/D	N/D	N/D	N/D	N/D	N/D	N/D	N/D	N/D	N/D	N/D	N/D	N/D	N/D	N/D
Succinate	0.10	0.92	0.21	0.57	0.04	0.57	0.01	1.55	0.06	0.59	0.02	0.04	0.02	0.62	2.73	0.64	0.08	0.96	35.39	1.04	0.03
Threonine	0.24	0.15	0.01	0.10	0.01	0.17	0.00	0.12	0.01	0.13	0.00	0.22	0.04	1.15	1.17	0.89	2.16	1.30	0.54	0.77	1.84
Tryptophan	0.04	0.02	N/D	0.02	N/D	0.02	0.00	0.02	0.00	0.02	0.00	0.01	0.00	1.20	0.83	1.11	0.52	1.09	1.59	0.92	0.63
Tyrosine	0.03	0.08	0.01	0.09	N/D	0.02	0.00	0.02	0.00	0.02	0.00	0.02	0.00	0.25	0.27	0.26	0.24	0.96	1.11	1.04	0.90
Valine	0.37	0.35	0.04	0.34	0.01	0.30	0.00	0.31	0.02	0.27	0.00	0.28	0.01	0.86	0.92	0.78	0.82	1.11	1.12	0.90	0.90

Table S2C - Chenomx Analysis of Δ*aphA* Δ*hapR* Δ*vpsL* strains

	Media (0 Hrs)		WT 8 Hrs		WT 19 Hrs		HCD 8 Hrs		HCD 19 Hrs		LCD 8 Hrs		LCD 19 Hrs		HCD/WT 8 Hrs	HCD/WT 19 Hrs	LCD/WT 8 Hrs	LCD/WT 19 Hrs	HCD/LCD 8 Hrs	HCD/LCD 19 Hrs	LCD/HCD 8 Hrs	LCD/HCD 19 Hrs
	[mM]	+/-	[mM]	+/-	[mM]	+/-	[mM]	+/-	[mM]	+/-	[mM]	+/-	[mM]	+/-	Fold Change	Fold Change	Fold Change	Fold Change	Fold Change	Fold Change	Fold Change	Fold Change
2,3 Butanediol	N/D	N/D	0.68	0.04	4.18	0.16	1.093	0.175	4.418	0.418	0.342	0.062	1.357	0.149	1.60	1.06	0.50	0.32	3.20	3.26	0.31	0.31
Acetate	0.089	0.011	3.57	0.07	3.42	0.16	3.391	0.107	1.814	0.065	6.013	0.488	7.313	0.604	0.95	0.53	1.68	2.14	0.56	0.25	1.77	4.03
Acetoin	N/D	N/D	0.35	0.04	0.98	0.04	0.729	0.150	1.620	0.488	0.261	0.065	0.263	0.056	2.07	1.66	0.74	0.27	2.79	6.16	0.36	0.16
Alanine	0.360	0.019	0.23	0.01	0.27	0.01	0.286	0.007	0.250	0.031	0.324	0.008	0.357	0.048	1.22	0.91	1.38	1.30	0.88	0.70	1.13	1.43
Aspartate	0.576	0.030	0.04	0.00	0.04	0.00	0.060	0.014	0.040	0.006	0.060	0.014	0.040	0.006	1.51	1.01	1.51	1.01	1.00	1.00	1.00	1.00
Ethanol	N/D	N/D	3.14	0.11	6.73	0.29	4.101	0.173	8.195	0.495	4.484	0.303	6.411	0.288	1.31	1.22	1.43	0.95	0.91	1.28	1.09	0.78
Formate	0.032	0.005	6.12	0.13	10.23	0.49	7.386	0.206	10.759	0.466	10.024	0.424	12.356	0.386	1.21	1.05	1.64	1.21	0.74	0.87	1.36	1.15
Glucose	28.374	1.052	14.76	0.16	1.58	0.42	16.552	1.047	6.482	N/D	17.467	0.338	9.943	0.885	1.12	4.11	1.18	6.30	0.95	0.65	1.06	1.53
Glutamate	0.979	0.074	0.74	0.03	0.74	0.05	0.680	0.153	0.744	0.049	0.665	0.149	0.567	0.158	0.92	1.00	0.90	0.76	1.02	1.31	0.98	0.76
Glycine	0.212	0.012	0.09	0.01	0.12	0.01	0.122	0.010	0.128	0.015	0.130	0.014	0.142	0.011	1.42	1.06	1.52	1.17	0.94	0.90	1.07	1.11
Isoleucine	0.203	0.008	0.16	0.00	0.17	0.02	0.216	0.006	0.217	0.012	0.216	0.007	0.228	0.002	1.39	1.27	1.39	1.34	1.00	0.95	1.00	1.06
Lactate	0.181	0.010	0.03	0.00	0.14	0.01	0.121	0.042	0.117	0.032	0.307	0.052	2.950	0.194	4.49	0.84	11.42	21.09	0.39	0.04	2.55	25.13
Leucine	0.498	0.025	0.31	0.05	0.39	0.02	0.474	0.012	0.425	0.032	0.459	0.050	0.433	0.026	1.51	1.08	1.46	1.10	1.03	0.98	0.97	1.02
Lysine	0.359	0.021	0.17	0.01	0.05	0.01	0.200	0.055	0.221	0.104	0.209	0.059	0.218	0.109	1.20	4.01	1.25	3.97	0.96	1.01	1.04	0.99
Methionine	0.116	0.014	0.09	0.00	0.10	0.01	0.136	0.017	0.140	0.018	0.136	0.017	0.135	0.017	1.43	1.37	1.43	1.32	1.00	1.04	1.00	0.96
Phenylalanine	0.157	0.004	0.11	0.00	0.13	0.01	0.130	0.015	0.147	0.009	0.147	0.009	0.147	0.009	1.23	1.17	1.40	1.17	0.88	1.00	1.14	1.00
Proline	0.581	0.036	0.41	0.02	0.38	0.04	0.473	0.048	0.418	0.068	0.367	0.063	0.399	0.064	1.16	1.10	0.90	1.05	1.29	1.05	0.78	0.96
Pyroglutamate	0.590	0.051	0.50	0.02	0.43	0.00	0.642	0.122	0.431	0.015	0.445	0.034	0.462	0.036	1.27	1.01	0.88	1.08	1.44	0.93	0.69	1.07
Pyruvate	N/D	N/D	0.19	0.03	0.01	0.00	0.063	N/D	0.051	0.011	0.796	0.154	0.850	0.139	0.34	3.53	4.27	58.59	0.08	0.06	12.64	16.58
Serine	0.389	0.027	N/D	N/D	N/D	N/D	N/D	N/D	N/D	N/D	N/D	N/D	N/D	N/D	N/D	N/D	N/D	N/D	N/D	N/D	N/D	N/D
Succinate	N/D	N/D	0.57	0.02	2.00	0.07	0.780	0.059	2.040	0.255	0.745	0.081	1.319	0.110	1.36	1.02	1.30	0.66	1.05	1.55	0.96	0.65
Threonine	0.239	0.023	0.14	0.01	0.07	0.03	0.104	0.033	0.083	0.038	0.135	0.061	0.071	0.020	0.75	1.21	0.97	1.04	0.77	1.16	1.30	0.86
Tyrosine	0.081	0.007	0.06	0.00	0.08	0.01	0.082	0.010	0.091	0.005	0.091	0.005	0.091	0.005	1.30	1.21	1.43	1.21	0.91	1.00	1.10	1.00
Valine	0.340	0.018	0.31	0.01	0.33	0.02	0.369	0.012	0.347	0.019	0.352	0.022	0.351	0.028	1.20	1.04	1.14	1.06	1.05	0.99	0.95	1.01

Table S2D - Bayesil Analysis of Δ*aphA* Δ*hapR* Δ*vpsL* strains

	Media (0 Hrs)		WT 8 Hrs		WT 19 Hrs		HCD 8 Hrs		HCD 19 Hrs		LCD 8 Hrs		LCD 19 Hrs		HCD/WT 8 Hrs	HCD/WT 19 Hrs	LCD/WT 8 Hrs	LCD/WT 19 Hrs	HCD/LCD 8 Hrs	HCD/LCD 19 Hrs	LCD/HCD 8 Hrs	LCD/HCD 19 Hrs
	[mM]	+/-	[mM]	+/-	[mM]	+/-	[mM]	+/-	[mM]	+/-	[mM]	+/-	[mM]	+/-	Fold Change	Fold Change	Fold Change	Fold Change	Fold Change	Fold Change	Fold Change	Fold Change
Acetate	0.04	0.01	1.10	0.03	1.06	0.04	2.24	0.04	1.28	0.07	4.04	0.30	2.53	1.14	2.03	1.21	3.67	2.39	0.55	0.51	1.81	1.98
Acetoacetate	0.01	0.01	N/D	N/D	N/D	N/D	N/D	N/D	0.01	0.01	N/D	N/D	N/D	N/D	N/D	N/D	N/D	N/D	N/D	N/D	N/D	N/D
Acetone	N/D	N/D	0.17	0.01	0.52	0.02	0.41	0.10	0.97	0.29	0.13	0.03	0.39	0.25	2.39	1.86	0.74	0.76	3.22	2.46	0.31	0.41
Alanine	0.22	0.02	0.11	0.00	0.13	0.01	0.18	0.01	0.16	0.02	0.24	0.01	0.24	0.03	1.61	1.26	2.23	1.89	0.72	0.67	1.38	1.50
Arginine	0.07	0.04	N/D	N/D	N/D	N/D	N/D	N/D	0.01	0.01	0.01	0.01	0.02	0.02	N/D	N/D	N/D	N/D	N/D	0.72	N/D	1.38
Asparagine	0.05	0.01	0.01	0.00	0.02	0.01	0.02	N/D	0.02	N/D	0.03	0.01	0.02	N/D	1.30	0.90	1.94	0.78	0.67	1.16	1.49	0.86
Aspartate	0.62	0.06	0.03	0.00	0.03	0.00	0.03	0.01	0.04	N/D	0.03	N/D	0.03	0.01	0.99	1.42	1.03	0.95	0.96	1.50	1.04	0.67
Ethanol	0.02	0.01	2.97	0.13	6.21	0.09	4.50	0.38	9.27	0.95	4.91	0.36	7.52	0.82	1.52	1.49	1.66	1.21	0.92	1.23	1.09	0.81
Formate	0.03	0.01	5.76	0.15	9.71	0.44	7.64	0.40	11.98	0.96	10.42	0.64	12.39	0.80	1.33	1.23	1.81	1.28	0.73	0.97	1.36	1.03
Glucose	28.77	2.46	12.78	0.36	1.06	0.33	15.04	0.32	1.43	1.41	15.83	0.39	6.23	2.15	1.18	1.34	1.24	5.87	0.95	0.23	1.05	4.37
Glutamic acid	1.15	0.06	1.46	0.05	1.65	0.08	1.04	0.05	1.15	0.14	0.91	0.02	0.81	0.08	0.71	0.70	0.63	0.49	1.14	1.42	0.87	0.71
Glutamine	0.07	0.02	0.02	0.01	0.07	0.04	0.02	0.02	0.11	0.06	0.03	0.02	0.17	0.12	1.37	1.53	1.99	2.49	0.68	0.61	1.46	1.63
Glycine	0.39	0.15	N/D	N/D	0.04	0.03	N/D	N/D	0.09	0.03	0.05	0.03	0.02	0.01	N/D	2.03	N/D	0.41	N/D	5.00	N/D	0.20
Histidine	0.03	0.01	0.11	0.01	0.07	0.01	N/D	N/D	0.01	N/D	N/D	N/D	0.01	N/D	N/D	0.08	N/D	0.09	N/D	0.84	N/D	1.19
Isoleucine	0.32	0.04	0.17	0.01	0.19	0.02	0.23	0.01	0.33	0.05	0.25	0.01	0.26	0.02	1.34	1.69	1.45	1.34	0.92	1.26	1.08	0.79
Lactate	0.06	0.05	0.12	0.01	0.20	0.02	0.09	0.03	0.10	0.05	0.34	0.04	1.71	0.55	0.77	0.50	2.91	8.37	0.26	0.06	3.78	16.81
Leucine	0.54	0.05	0.38	0.03	0.29	0.03	0.47	0.03	0.48	0.04	0.46	0.02	0.45	0.03	1.25	1.65	1.22	1.57	1.03	1.06	0.97	0.95
Lysine	0.32	0.03	0.22	0.02	0.45	0.02	0.32	0.02	0.54	0.03	0.34	0.05	0.48	0.05	1.42	1.18	1.51	1.06	0.94	1.12	1.07	0.89
Malonate	0.17	0.03	0.02	0.00	0.01	0.00	0.02	N/D	0.01	N/D	0.02	N/D	0.01	N/D	1.11	0.97	1.33	1.85	0.84	0.52	1.19	1.91
Methionine	0.11	0.03	0.09	0.01	0.10	0.00	0.12	0.01	0.11	0.01	0.12	0.01	0.12	0.01	1.26	1.14	1.27	1.18	0.99	0.96	1.01	1.04
Phenylalanine	0.18	0.02	0.11	0.00	0.12	0.01	0.14	N/D	0.16	0.02	0.15	0.01	0.15	0.01	1.28	1.32	1.34	1.24	0.96	1.06	1.04	0.94
Proline	0.66	0.11	0.25	0.01	0.33	0.03	0.46	0.02	0.45	0.07	0.26	0.03	0.15	0.06	1.89	1.37	1.06	0.46	1.78	2.99	0.56	0.33
Pyruvic acid	N/D	N/D	0.19	0.02	N/D	N/D	0.01	N/D	0.01	N/D	0.78	0.13	0.61	0.24	0.07	N/D	4.16	N/D	0.02	0.01	62.47	71.09
Serine	1.54	0.24	0.02	0.01	0.02	0.02	0.02	0.01	0.01	0.01	N/D	N/D	0.01	0.01	0.79	0.61	N/D	0.56	N/D	1.08	N/D	0.92
Succinate	0.11	0.01	0.61	0.02	2.10	0.07	0.90	0.08	2.42	0.32	0.67	0.06	1.03	0.61	1.48	1.15	1.10	0.49	1.34	2.34	0.74	0.43
Threonine	0.25	0.02	0.13	0.00	0.06	0.01	0.17	0.01	0.08	0.02	0.16	N/D	0.24	0.08	1.31	1.27	1.29	3.77	1.02	0.34	0.98	2.96
Tryptophan	0.06	0.02	0.02	0.00	0.01	0.00	0.01	N/D	0.02	0.01	0.02	N/D	0.02	0.01	0.80	2.29	1.03	1.59	0.77	1.44	1.29	0.70
Tyrosine	0.09	0.01	0.02	0.00	0.02	0.00	0.08	N/D	0.09	0.01	0.09	N/D	0.09	N/D	4.51	3.81	4.98	3.98	0.91	0.96	1.10	1.05
Valine	0.37	0.03	0.27	0.01	0.26	0.04	0.40	0.02	0.44	0.06	0.37	0.02	0.34	0.05	1.50	1.68	1.38	1.30	1.09	1.29	0.92	0.77
